# Novel Dent disease 1 cellular models reveal biological processes underlying ClC-5 loss-of-function

**DOI:** 10.1093/hmg/ddab131

**Published:** 2021-05-13

**Authors:** Mónica Durán, Carla Burballa, Gerard Cantero-Recasens, Cristian M Butnaru, Vivek Malhotra, Gema Ariceta, Eduard Sarró, Anna Meseguer

**Affiliations:** Renal Physiopathology Group, Vall d'Hebron Research Institute (VHIR)-CIBBIM Nanomedicine, Barcelona, Spain; Renal Physiopathology Group, Vall d'Hebron Research Institute (VHIR)-CIBBIM Nanomedicine, Barcelona, Spain; Centre for Genomic Regulation, The Barcelona Institute of Science and Technology, Barcelona, Spain; Renal Physiopathology Group, Vall d'Hebron Research Institute (VHIR)-CIBBIM Nanomedicine, Barcelona, Spain; Centre for Genomic Regulation, The Barcelona Institute of Science and Technology, Barcelona, Spain; Centre for Genomic Regulation, The Barcelona Institute of Science and Technology, Barcelona, Spain; Institució Catalana de Recerca i Estudis Avançats, Barcelona, Spain; Renal Physiopathology Group, Vall d'Hebron Research Institute (VHIR)-CIBBIM Nanomedicine, Barcelona, Spain; Pediatric Nephrology Department, Vall d'Hebron University Hospital, Universitat Autònoma de Barcelona, Barcelona, Spain; Renal Physiopathology Group, Vall d'Hebron Research Institute (VHIR)-CIBBIM Nanomedicine, Barcelona, Spain; Renal Physiopathology Group, Vall d'Hebron Research Institute (VHIR)-CIBBIM Nanomedicine, Barcelona, Spain; Departament de Bioquímica i Biologia Molecular, Unitat de Bioquímica de Medicina, Universitat Autònoma de Barcelona (UAB), Bellaterra, Spain; Red de Investigación Renal (REDINREN), Instituto de Salud Carlos III-FEDER, Madrid, Spain

## Abstract

Dent disease 1 (DD1) is a rare X-linked renal proximal tubulopathy characterized by low molecular weight proteinuria and variable degree of hypercalciuria, nephrocalcinosis and/or nephrolithiasis, progressing to chronic kidney disease. Although mutations in the electrogenic Cl^−^/H^+^ antiporter ClC-5, which impair endocytic uptake in proximal tubule cells, cause the disease, there is poor genotype–phenotype correlation and their contribution to proximal tubule dysfunction remains unclear. To further discover the mechanisms linking ClC-5 loss-of-function to proximal tubule dysfunction, we have generated novel DD1 cellular models depleted of ClC-5 and carrying ClC-5 mutants p.(Val523del), p.(Glu527Asp) and p.(Ile524Lys) using the human proximal tubule-derived RPTEC/TERT1 cell line. Our DD1 cellular models exhibit impaired albumin endocytosis, increased substrate adhesion and decreased collective migration, correlating with a less differentiated epithelial phenotype. Despite sharing functional features, these DD1 cell models exhibit different gene expression profiles, being p.(Val523del) ClC-5 the mutation showing the largest differences. Gene set enrichment analysis pointed to kidney development, anion homeostasis, organic acid transport, extracellular matrix organization and cell-migration biological processes as the most likely involved in DD1 pathophysiology. In conclusion, our results revealed the pathways linking ClC-5 mutations with tubular dysfunction and, importantly, provide new cellular models to further study DD1 pathophysiology.

## Introduction

Dent disease 1 (DD1; OMIM #300009) is a rare X-linked renal tubulopathy affecting about 330 families world-wide (1) and characterized by low molecular weight proteinuria (LMWP), and variable degree and occurrence of hypercalciuria, nephrocalcinosis, nephrolithiasis and hypophosphatemic rickets (2,3). DD1 progresses to renal failure between the third and fifth decades of life in 30–80% of affected males, while female carriers are usually asymptomatic (4). Progression to CKD in DD1 is associated with decreasing calcium excretion in urine, which normalizes in 40% of affected adults < 30 years, and in 85% of those >30 years of age (5). Lack of hypercalciuria, non-specific manifestations, overlap with glomerular disorders (6) and other genetic forms of renal Fanconi syndrome with overt LMWP (7) may explain common underdiagnosis of DD1 which accurate prevalence is unknown. There is no current curative treatment for DD1, nor clinical trials ongoing, and patient’s care is supportive, focusing on the treatment of hypercalciuria and the prevention of nephrolithiasis (8). DD1 is caused by loss-of-function mutations in the *CLCN5* gene encoding the electrogenic 2 Cl^−^/H^+^ antiporter ClC-5, which is abundantly expressed in ﻿the epithelia of kidney and intestine, though it is also expressed in brain, lung and, to a lesser extent, liver (9). In the human kidney, ClC-5 is mainly expressed in proximal tubule cells (PTCs), where it is predominantly located in intracellular subapical endosomes and participates in endosomal acidification (2,10). A small fraction of ClC-5 is also found on the plasma membrane of PTCs, where it is proposed to mediate plasma membrane chloride currents (10) or participate in the macromolecular complexes responsible for low molecular weight (LMW) protein and albumin endocytosis (11).

PTCs reabsorb ~65% of filtered load and most of filtered LMW proteins mainly via receptor-mediated endocytosis (12). The main actor in LMW protein reabsorption is the endocytic complex, which is comprised by the multiligand tandem receptors megalin and cubilin. Receptor-mediated endocytosis requires a continuous cycling of megalin and cubilin between the apical plasma membrane, where they specifically bind ultrafiltrated LMW proteins and other ligands, and the early endosome, where the receptors dissociate from their bound ligands (13). This process requires vesicular acidification for dissociating the ligand-receptor complex, recycling of receptors to the apical membrane and progression of ligands into the lysosomes. Endosomal acidification is achieved by ATP-driven transport of cytosolic H^+^ through the vacuolar H^+^-ATPase (14). Inactivating mutations of *CLCN5* in Dent disease patients (15) as well as the deletion of *CLCN5* in knock-out (KO) mice (16,17) lead to severe LMWP due to a defective endocytic uptake in PTCs, which has been associated with the disappearance of megalin and cubilin at the brush border of PTCs. ClC-5 was initially postulated to provide a Cl^−^ shunt into the lumen of endosomes to dissipate V-ATPase-mediated H^+^ accumulation, thereby enabling efficient endosomal acidification (14,18). However, mutations in ClC-5 causing Dent disease do not necessarily lead to a defective endosomal acidification (19), suggesting that the disease may result from an impaired exchange activity, namely uncoupling Cl^−^/H^+^ co-transport and altered Cl^−^ accumulation at early endosomes (20). Thus, the precise molecular role of ClC-5 in endosomal physiology and endocytosis, as well as several aspects of its ion transport properties remain to be fully elucidated.

To date, a total of 266 pathogenic variants of *CLCN5* have been reported consisting of nonsense, missense, splice site, insertion and deletion mutations (1,21). According to the latest reports, *CLCN5* mutations are grouped into three classes on the basis of functional data (19,21,22): class 1 mutations result in defective protein processing and folding, thereby inducing retention of the mutant protein in the endoplasmic reticulum (ER), where they are early degraded by quality control systems; class 2 mutations impair protein processing and stability, leading to a functionally defective protein lacking electric currents; these mutants show reduced expression in the plasma membrane, but a normal distribution in the early endosomes; and class 3 mutations generate a protein that reaches the plasma membrane and early endosomes correctly, but shows reduced or abolished currents. Yet, very little is known regarding how these mutations lead to specific disease manifestations. In this sense, the considerable intra-familial variability in disease severity and the lack of genotype–phenotype correlation suggest that unknown mechanisms might be involved in PTCs dysfunction leading to DD1 progression. In order to identify these ClC-5 mutation-associated pathways, we have knock down the *CLCN5* gene or introduced the ClC-5 mutations p.(Val523del) (not classified), p.(Glu527Asp) (class 2) or p.(Ile524Lys) (class 1) in RPTEC/TERT1 cells, producing specific cellular models carrying exogenous wild-type (WT) or mutant ClC-5 proteins. The parental cell line represents one of the most well-differentiated and stable human proximal tubular cell line currently available, retaining sodium-dependent phosphate uptake and an intact functionality of the megalin–cubilin transport system (23,24). Gene expression profiling and functional analysis in these newly created DD1 cells revealed the biological processes (BP) related to proximal tubule dysfunction in DD1, likely explaining phenotype variability of the disease and the progression to renal failure.

## Results

### Expression and subcellular localization of ClC-5 mutants p.(Val523del), p.(Glu527Asp) and p.(Ile524Lys) in RPTEC/TERT1 cells

**Figure 1:**
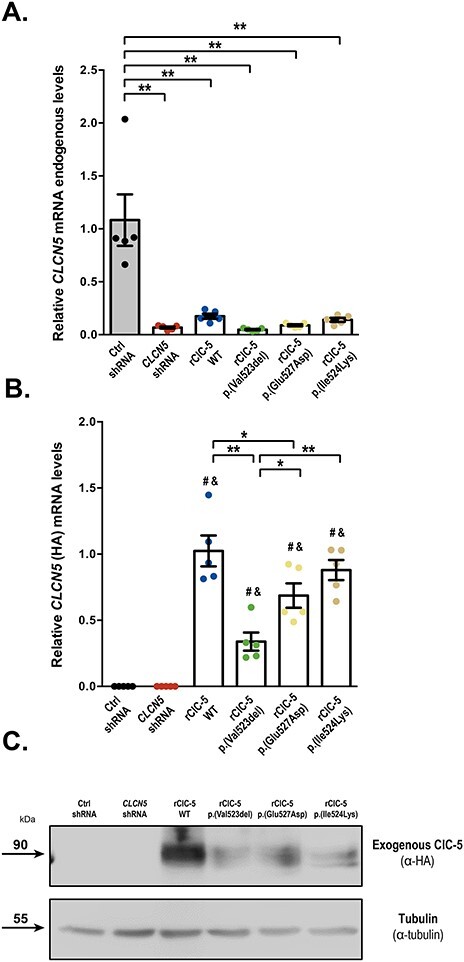
Expression levels of ClC-5 mutants p.(Val523del), p.(Glu527Asp) and p.(Ile524Lys) in RPTEC/TERT1 cells. (**A**) mRNA levels of endogenous *CLCN5* from control cells (Ctrl), *CLCN5*-silenced cells (*CLCN5* shRNA), and *CLCN5*-silenced cells carrying WT (rClC-5 WT) and mutant (rClC-5 p.(Val523del), rClC-5 p.(Glu527Asp) and rClC-5 p.(Ile524Lys)) ClC-5 were measured by RT-qPCR using specific probes targeting the intact shRNA target sequence. (**B**) The expression levels of re-introduced WT (rClC-5 WT) and mutant (rClC-5 p.(Val523del), rClC-5 p.(Glu527Asp) and rClC-5 p.(Ile524Lys)) ClC-5 were assessed by RT-qPCR using specific probes against the HA-tag, which is only present in the exogenous ClC-5. (**C**) Protein levels of re-introduced WT and mutant ClC-5 were analyzed by western blot using an antibody against the HA-tag. Tubulin was used to ensure that equal amounts of total cell extract were loaded in each lane. For all experiments, Ctrl shRNA corresponds to cells transduced with both shRNA empty vector and re-expression empty vector. Dots represent individual values and columns indicate the mean ± SEM, *n* > 3. Statistical significance was determined using One-way ANOVA followed by Tukey’s multiple comparisons *post hoc* test. ^*^, *P* < 0.05; ^*^^*^, *P* < 0.01. # *P* < 0.01 compared with Ctrl shRNA; & *P* < 0.01 compared with *CLCN5* shRNA.

To explore the molecular mechanisms underlying PTCs dysfunction in DD1, we first generated stable RPTEC/TERT1 cell lines silenced for *CLCN5* gene or carrying the pathogenic ClC-5 mutations p.(Val523del), p.(Glu527Asp) or p.(Ile524Lys) (described in DD1 patients (25–27)). A scheme summarizing the generation of the RPTEC/TERT1 DD1 cell model and the localization of shRNA sequences and mutations in the corresponding ClC-5 protein is provided in Figures S1A and B, respectively. First, to fully characterize these cell lines, RNA was extracted from 10-day differentiated control (Ctrl shRNA), knockdown (*CLCN5* shRNA), and knockdown cells carrying the HA-tagged recombinant WT (rClC-5 WT), or mutant ClC-5 forms p.(Val523del), p.(Glu527Asp) and p.(Ile524Lys), to assess the levels of endogenous, exogenous (HA-tagged) and total *CLCN5* levels by means of quantitative real-time PCR (RT-qPCR). Our results showed that endogenous mRNA levels of *CLCN5* were strongly reduced in all cell lines transduced with the shRNA against *CLCN5*, compared with control cells (Ctrl shRNA): *CLCN5* shRNA (6.2%, *P* < 0.0001 vs Ctrl shRNA), rClC-5 WT (16.1%, *P* < 0.0001 vs Ctrl shRNA), rClC-5 p.(Val523del) (4.4%, *P* < 0.0001 vs Ctrl shRNA), rClC-5 p.(Glu527Asp) (8.2%, *P* < 0.0001 vs Ctrl shRNA) and rClC-5 p.(Ile524Lys) (13%, *P* < 0.0001 vs Ctrl shRNA) (Fig. 1A). Re-introduction of HA-tagged WT (rClC-5 WT) and mutant (rClC-5 p.(Val523del), rClC-5 p.(Glu527Asp) and rClC-5 p.(Ile524Lys)) forms in former silenced cells restored *CLCN5* mRNA levels, as assessed with RT-qPCR probes against the HA tag (Fig. 1B) and total *CLCN5* (Supplementary Material, Fig. S1C), although mutants rClC-5 p.(Val523del) (33.8%, *P* < 0.0001 vs WT) and rClC-5 p.(Glu527Asp) (68.7%, *P* = 0.0372 vs rClC-5 WT), but not rClC-5 p.(Ile524Lys) (87.9%, *P* = 0.7362 vs rClC-5 WT) exhibited lower exogenous *CLCN5* levels than those of rClC-5 WT cells. At the protein level, ClC-5 was detected as a lower band running at 80–90 kDa and a higher diffuse band running as a smear at about 100 kDa, which was consistent with previous reports (28,29) (Fig. 1C). Loading equivalent amount of cell extract revealed that the protein levels of all the three ClC-5 mutants were strongly reduced in comparison with rClC-5 WT (Fig. 1C).

**Figure 2:**
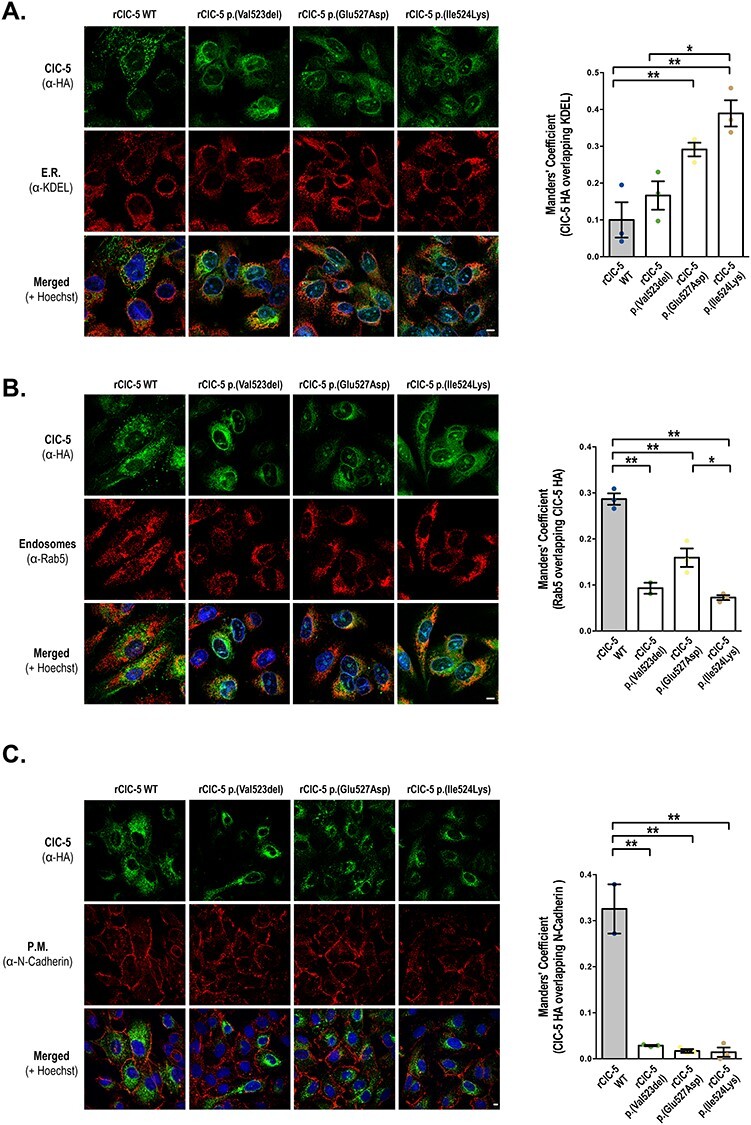
p.(Val523del), p.(Glu527Asp) and p.(Ile524Lys) mutations alter the subcellular localization of ClC-5 in RPTEC/TERT1 cells. Subcellular localization of WT (rClC-5 WT) and mutant (rClC-5 p.(Val523del), rClC-5 p.(Glu527Asp) and rClC-5 p.(Ile524Lys)) ClC-5 was analyzed in RPTEC/TERT1 cells seeded on glass coverslips by determining their co-localization with the ER marker KDEL (**A**), early endosomes (EE) marker Rab-5 (**B**) and plasma membrane (PM) marker N-cadherin (**C**), using the corresponding antibodies. Cell nuclei were stained with Hoescht. Scale bars correspond to 5 μm. Quantification of the co-localization was performed using the Manders’ overlap coefficient (MOC). Dots represent individual values and columns indicate the mean ± SEM, *n* = 3 (minimum five cells/field). Statistical significance was determined using One-way ANOVA followed by Tukey’s multiple comparisons *post hoc* test. ^*^, *P* < 0.05; ^*^^*^, *P* < 0.01. Abbreviations: E.R., endoplasmic reticulum; P.M., plasma membrane.

We next analyzed the subcellular localization of WT and mutant ClC-5 in RPTEC/TERT1 cells by using immunostaining techniques. Our results show that rClC-5 WT localized at the plasma membrane (P.M.), early endosomes (E.E.) and endoplasmic reticulum (E.R.), as shown by co-localization with the specific subcellular compartment markers N-cadherin (P.M.), Rab-5 (E.E.) and KDEL (E.R.) (Fig. 2A, B and C). Quantification of the co-localization of ClC-5 forms with KDEL using the Manders’ overlap coefficient (MOC) demonstrated that rClC-5 p.(Glu527Asp) (MOC = 0.29, *P* = 0.0256 vs rClC-5 WT) and rClC-5 p.(Ile524Lys) (MOC = 0.39, *P* = 0.0024 vs rClC-5 WT), but not rClC-5 p.(Val523del) (MOC = 0.17, *P* = 0.6005 vs rClC-5 WT) accumulated at the ER to a greater extent than rClC-5 WT (MOC = 0.10). Moreover, all three mutants showed a reduced co-localization with Rab5 (MOC rClC-5 WT = 0.29, rClC-5 p.(Val523del) = 0.09 (*P* = 0.0002 vs rClC-5 WT), rClC-5 p.(Glu527Asp) = 0.16 (*P* = 0.0012 vs rClC-5 WT) and rClC-5 p.(Ile524Lys) = 0.07 (*P* < 0.0001 vs rClC-5 WT)) and N-cadherin (MOC rClC-5 WT = 0.33, rClC-5 p.(Val523del) = 0.03 (*P* < 0.0001 vs rClC-5 WT), rClC-5 p.(Glu527Asp) = 0.02 (*P* < 0.0001 vs rClC-5 WT) and rClC-5 p.(Ile524Lys) = 0.01 (*P* < 0.0001 vs rClC-5 WT)) in comparison with rClC-5 WT, indicating that their presence in E.E. and P.M. was reduced. These results confirmed, in the case of rClC-5 p.(Glu527Asp) and rClC-5 p.(Ile524Lys) subcellular localizations, previous results in HEK-MSR cells (19).

### ClC-5 p.(Ile524Lys) mutant, but not p.(Val523del) or p.(Glu527Asp) presents an altered glycosylation pattern

**Figure 3:**
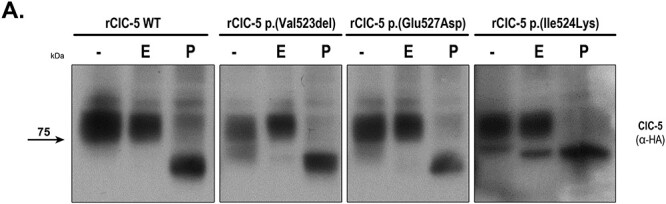
ClC-5 mutant p.(Ile524Lys), but not p.(Val523del) or p.(Glu527Asp), presents an altered glycosylation pattern. To explore the effects of selected ClC-5 mutations on the glycosylation pattern of ClC-5, total cell lysates from RPTEC/TERT1 cells carrying each of the mutations were treated with Endoglycosidase H (E), which cleaves asparagine-linked mannose rich oligosaccharides, but not highly processed complex oligosaccharides, and Peptide:N-glycosidase F (PNGase F) (P), which cleaves between the innermost GlcNAc and asparagine residues of high mannose, hybrid, and complex oligosaccharides. After glycosidase reactions, samples were analyzed by western blot using an anti HA-tag antibody.

It has been previously described that ClC-5 undergoes several post-translational modifications, including glycosylation (30). Moreover, mutations on ClC-5 N-glycosylation sites trigger poly-ubiquitination and proteasomal degradation (29,30). To gain more insight on whether the differences in protein levels and subcellular localization between rClC-5 WT and ClC-5 mutants could be related to impaired glycosylation processing, cell lysates from each of the conditions were treated with Endoglycosidase H (Endo H), which cleaves asparagine-linked mannose rich oligosaccharides, but not highly processed complex oligosaccharides, and Peptide:N-glycosidase F (PNGase F), which cleaves between the innermost GlcNAc and asparagine residues of high mannose, hybrid, and complex oligosaccharides. Our results show that rClC-5 WT and all mutant forms of ClC-5 were sensitive to PNGase F digestion, confirming that all of them were N-glycosylated (Fig. 3). On the other hand, only the lower migrating band of p.(Ile524Lys) mutant was sensible to EndoH digestion, as observed by a reduction in its molecular weight. These results suggest that this faster migrating band of the p.(Ile524Lys) mutant might correspond to a core-glycosylated EndoH-sensitive form of the protein, correlating with the higher degree of ER retention observed for this mutant.

To investigate whether the expression of ClC-5 mutants, and more specifically, p.(Ile524Lys), could be inducing the unfolded protein response (UPR) and ER stress as a result of their accumulation in the ER, we checked the phosphorylation of the ER stress marker PERK (31) (Supplementary Material, Fig. S2A) and the cleavage of XBP-1 mRNA (31) (Supplementary Material, Fig. S2B) in cells expressing WT or mutant ClC-5. Our results show that neither expression of rClC-5 WT nor any of the ClC-5 mutants studied induced detectable levels of ER stress.

### *CLCN5* silencing or re-introduction of p.(Val523del), p.(Glu527Asp) and p.(Ile524Lys) ClC-5 mutants impairs albumin endocytosis

**Figure 4:**
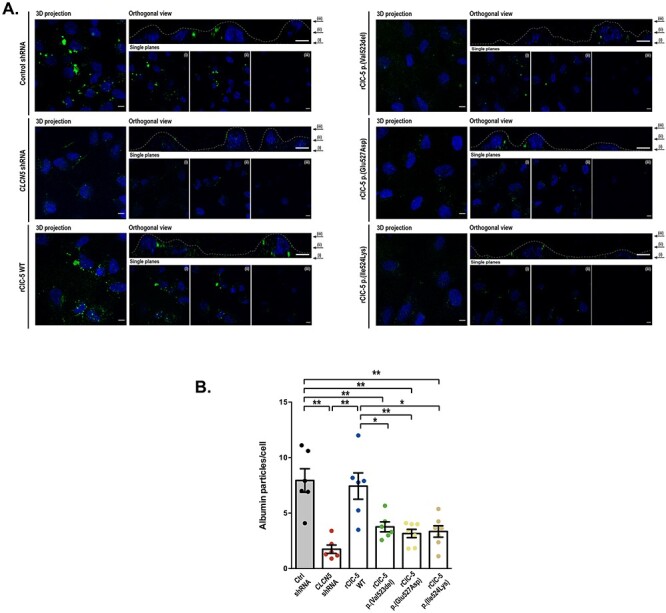
*CLCN5* silencing or p.(Val523del), p.(Glu527Asp) and p.(Ile524Lys) ClC-5 mutations impair Albumin endocytosis. (**A**) Control, *CLCN5* shRNA, rClC-5 WT, rClC-5 p.(Val523del), rClC-5 p.(Glu527Asp) and rClC-5 p.(Ile524Lys) cells were seeded on glass coverslips and incubated with 50 μg/mL Alexa Fluor 488-conjugated Albumin for 60 min. In the orthogonal view, dotted lines across the images demarcate the top surface of the cell. Scale bars correspond to 5 μm. (**B**) Quantification of albumin uptake was performed by measuring the number of albumin particles/cell. Dots represent individual values and columns indicate the mean ± SEM, *N* (number of experiments) > 3, *n* (number of cells) > 30. Outliers were removed using the Grubb’s test (alpha = 0.05). Statistical significance was determined using One-way ANOVA followed by Tukey’s multiple comparisons *post hoc* test. ^*^, *P* < 0.05; ^*^^*^, *P* < 0.01.

To determine the effect of *CLCN5* silencing and the selected ClC-5 mutations on the endocytic capacity of RPTEC/TERT1 cells, we analyzed Alexa Fluor 488-labelled albumin uptake. Detection of labeled albumin within the cell boundaries, both in orthogonal views and single planes, demonstrated that the endocytic machinery functions properly in the RPTEC/TERT1 cell line (Fig. 4A). *CLCN5* silencing strongly reduced the uptake of labelled albumin, whereas the endocytic capacity was re-established when the WT ClC-5 was re-introduced into the silenced cells (number of albumin Particles/cell Ctrl shRNA = 7.23, *CLCN5* shRNA = 2.27 (P = 0.0065 vs Ctrl shRNA) and rClC-5 WT = 6.57 (*P* = 0.6942 vs Ctrl shRNA) (Fig. 4A and B). By contrast, none of the ClC-5 mutants were able to restore this activity (number of albumin particles/cell rClC-5 p.(Val523del) = 3.15 (*P* = 0.0304 vs Ctrl shRNA), rClC-5 p.(Glu527Asp) = 3.7 (*P* = 0.0350 vs Ctrl shRNA) and rClC-5 p.(Ile524Lys) = 2.58 (*P* = 0.0093 vs Ctrl shRNA)), what indicates that these residues are essential for ClC-5-mediated endocytosis (Fig. 4A and B).

### *CLCN5* silencing or re-introduction of ClC-5 mutations p.(Val523del), p.(Glu527Asp) and p.(Ile524Lys) alter the global gene expression profile of RPTEC/TERT1 cells

**Figure 5:**
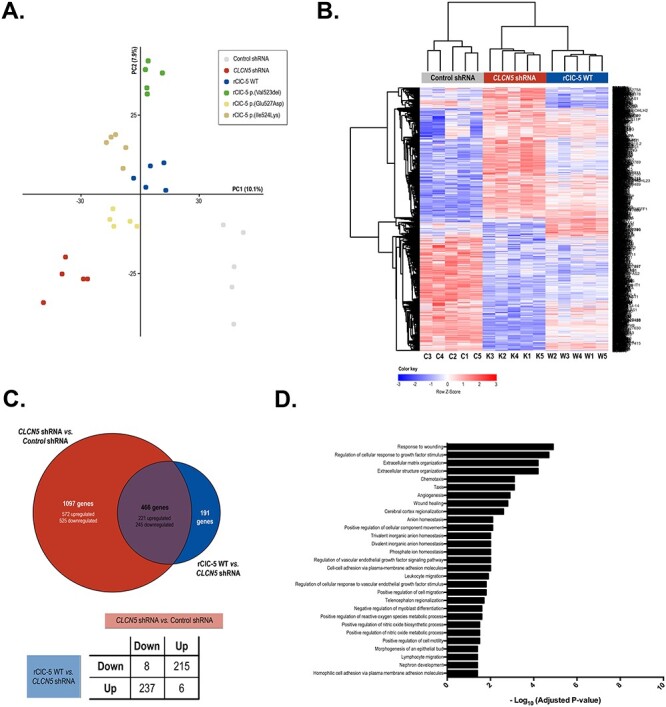
*CLCN5* silencing alters the global gene expression profile of RPTEC/TERT1 cells. To identify the potential mechanisms involved in the proximal tubule dysfunction secondary to the loss of ClC-5, we analyzed the gene expression profile of RPTEC/TERT1 cells carrying *CLCN5* silencing or rClC-5 WT using DNA microarrays. (**A**) PCA obtained after normalization and batch effect corrections of the DNA microarray data. (**B**) Heatmap graphically illustrating the differences in the expression levels of the 1754 genes that were up- or down-regulated with a logFC > |0.5| and an adj.p.val < 0.05 in *CLCN5*-silenced cells (*CLCN5* shRNA) in comparison to control cells (control shRNA) and *CLCN5*-silenced cells where WT ClC-5 was re-introduced (rClC-5 WT). (**C**) Venn diagrams depicting the genes commonly regulated by the effect of *CLCN5* silencing and rClC-5 WT re-introduction. The table indicates the number of common genes that are up- or down-regulated in each comparison. (**D**) Analysis of over-represented GO terms BPs related to the 452 genes that were commonly and oppositely up- or down-regulated by the effect of *CLCN5* silencing and rClC-5 WT re-introduction with a logFC > |0.5| and an adj.p.val < 0.05, using the GProfiler server.

In order to discover the putative mechanisms involved in proximal tubule dysfunction secondary to the loss of ClC-5, the gene expression profile of DD1 cell models was analyzed using DNA microarrays. To make the data comparable, as well as to remove technical biases, microarray data were first normalized and batch effect corrected. The principal component analysis (PCA) shown in Figure 5A, indicated that samples from the same experimental condition were aggregated. In addition, and to assess the reliability of the microarray data, the expression levels of *EMX2*, *PTPRD*, *STEAP1*, *ZPLD1*, *CDH1*, *NR1H4* and *EHF* genes were analyzed by qRT-PCR (Supplementary Material, Fig. S3). Validation genes were selected among those that met the following requirements: (i) altered expression between the WT condition and any of the mutations studied, and (ii) modified expression by *CLCN5* silencing and total or partial recovery upon ClC-5 WT re-introduction. Our results show that the validation gene selected panel presented an expression pattern consistent to that found in the DNA microarray (Supplementary Material, Fig. S3), thereby confirming the trustworthiness of the microarray data. For comparative analysis, genes were defined as differentially expressed genes (DEGs) if they presented an adjusted *P* value lower than 0.05 and a log2 Fold Change (logFC) higher or equal to 0.5 in any of the comparisons studied. Top up- and down-regulated DEGs in each of the comparisons are shown in Supplementary Material, Tables S1–S8.

The heatmap in Figure 5B graphically illustrates the differences in the gene expression profile of *CLCN5*-silenced cells in comparison with control cells and *CLCN5*-silenced cells with re-introduced WT ClC-5. These results show that *CLCN5* silencing elicited a marked effect on the gene expression profile of RPTEC/TERT1 cells, and that this effect was partially reversed by re-introducing the rClC-5 WT (Fig. 5B). Notably, from the 1563 genes altered by *CLCN5* silencing, up to 452 (from a total of 466 commonly regulated genes) were regulated in the opposite direction by *CLCN5* re-introduction (Fig. 5C). Among these, 237 (52%) genes were down-regulated and 215 (48%) were up-regulated by the effect of *CLCN5* silencing. Only the genes that were commonly regulated by *CLCN5* silencing and rClC-5 WT re-introduction (452 genes) were considered for subsequent analysis. To study in which BP were involved these genes, we performed an analysis of over-represented gene ontology (GO) terms. A list of the BP GO terms significantly enriched is shown in Figure 5D. We identified GO terms related to (intersection size/term size): response to wounding (36/372), matrix organization (27/397), anion homeostasis (5/65), cell adhesion (16/275), cell migration (29/569), positive regulation of reactive oxygen species (ROS) (12/107) and nephron development (13/146), among others. In anion homeostasis, significantly changed transcripts were *SLC34A2*, *NR1H4*, *TFAP2B*, *SFRP4* and *SLC7A11*, and in nephron development were *ADAMTS16*, *TFAP2B*, *NOG*, *FMN1*, *LGR4*, *DLL1*, *COL4A4*, *KIF26B*, *SULF1*, *NID1*, *TACSTD2*, *PROM1* and *BMP4*.

**Figure 6:**
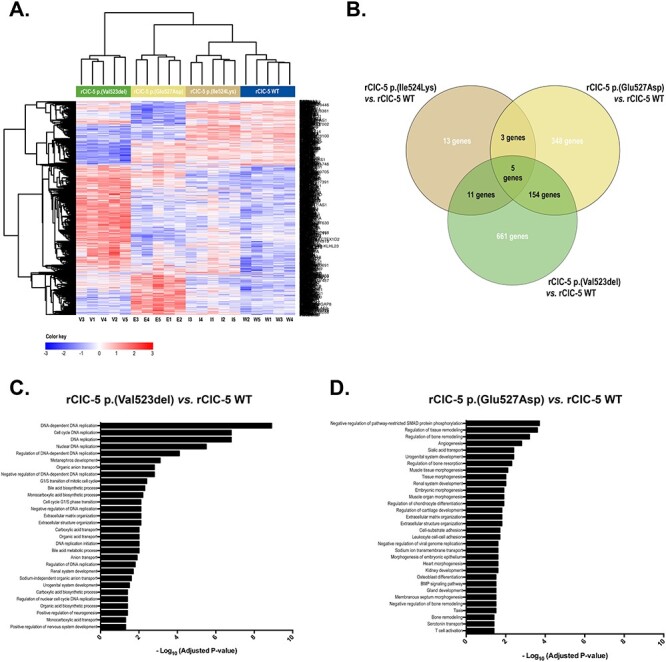
ClC-5 mutations p.(Val523del), p.(Glu527Asp) and p.(Ile524Lys) exert different effects on RPTEC/TERT1 gene expression profile. In order to explore the effects of the selected ClC-5 mutations on the transcriptome of RPTEC/TERT1 cells, gene expression profiles of p.(Val523del), p.(Glu527Asp) and p.(Ile524Lys) mutants were compared to that of WT ClC-5. (**A**) Heatmap showing the expression levels of the 1195 genes that were up-or down-regulated with a logFC > |0.5| and an adj.p.val < 0.05 in *CLCN5*-silenced cells where p.(Val523del), p.(Glu527Asp) or p.(Ile524Lys) ClC-5 was re-introduced in comparison to *CLCN5*-silenced cells where WT ClC-5 was re-introduced (rClC-5 WT). This results show that p.(Val523del) and p.(Ile524Lys) were the conditions that exhibited the greatest and the smallest differences in the gene expression profile, respectively, when compared to WT ClC-5. (**B**) Venn diagrams depicting the number of commonly regulated genes by ClC-5 mutations. Only five genes were shown to be commonly affected by all three mutations. GProfiler analysis of the significantly enriched BPs associated with the 831 genes up- or down-regulated with a logFC > |0.5| and adj.p.val < 0.05 by p.(Val523del) mutation (**C**), or associated with the 510 genes up- or down-regulated with a logFC > |0.5| and adj.p.val < 0.05 by p.(Glu527Asp) mutation (**D**), in comparison to ClC-5 WT.

In order to explore the effects of the selected ClC-5 mutations on the transcriptome of RPTEC/TERT1 cells, gene expression profiles of p.(Val523del), p.(Glu527Asp) and p.(Ile524Lys) mutants were compared to that of WT ClC-5. As shown in the heatmap in Figure 6A, p.(Val523del) and p.(Ile524Lys) were the conditions that exhibited the greatest and the smallest differences in the gene expression profile, respectively, when compared to WT ClC-5. Moreover, only five genes (*CHCHD7*, *PREX1*, *PLAG1*, *LIN54* and *ZRANB3*) were commonly affected by all three mutations (Fig. 6B). Re-introduction of p.(Val523del) mutant altered the expression of 831 genes compared to ClC-5 WT cells, from which 231 were down-regulated and 600 were up-regulated. Roughly 20% (47/231) of the down- and 5% (27/600) of the up-regulated genes by Val523del expression were also found down- or up-regulated, respectively, in *CLCN5*-silenced cells, and might represent genes whose expression levels cannot be restored by p.(Val523del) mutant to the levels achieved by WT ClC-5. On the other hand, genes altered by p.(Val523del) that were not affected in *CLCN5*-silenced cells could be indicating a gain of functionality of this mutant with regard to *CLCN5* silencing. An analysis of the significantly enriched BPs (GO terms) showed that p.(Val523del) altered the expression of genes mainly related to DNA replication, but also related to carboxylic acid and anion transport and renal system development, among others (Fig. 6C). ClC-5 mutant p.(Glu527Asp) altered the expression of 510 genes (204 down-regulated and 306 up-regulated). Among them, 24% of the down- (49/204) and 16% (48/306) of the up-regulated genes were also found down- or up-regulated, respectively, by effect of *CLCN5* silencing. Genes whose expression was significantly altered by p.(Glu527Asp) in comparison to WT ClC-5 were specially enriched in processes related to tissue remodeling and morphogenesis, but also in other processes including (intersection size/term size): sialic acid transport (3/7), sodium ion transmembrane transport (8/152) and also renal system development (19/315), for example (Fig. 6D). Finally, p.(Ile524Lys) ClC-5 mutation altered the expression of only 32 genes compared to ClC-5 WT, with 6 of them down-regulated and 26 up-regulated. This low number of genes did not allow to obtain any significantly enriched GO term in the over-representation analysis. Moreover, it might also account for the low number of common genes found at the intersection of the three mutant models.

### *CLCN5* silencing and ClC-5 mutations downregulate epithelial markers expression, increase cell proliferation and impair cell-to-substrate adhesion and collective cell migration

**Figure 7:**
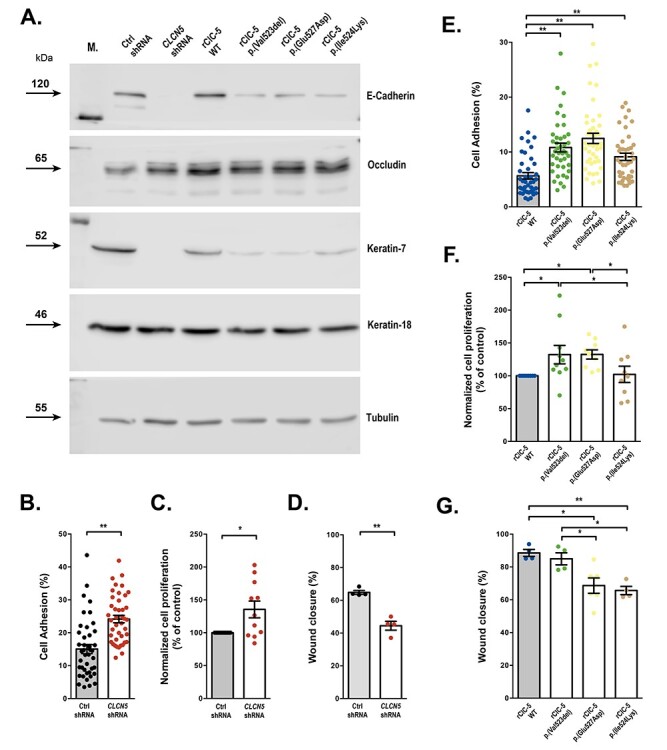
*CLCN5* silencing and mutations p.(Val523del), p.(Glu527Asp) and p.(Ile524Lys) impair cell-to-substrate adhesion and collective cell migration. To explore whether the changes observed in the gene expression profiles by *CLCN5* silencing or loss-of-function ClC-5 mutations correlated with changes in the epithelial characteristics, we analyzed substrate adhesion, proliferation and collective cell migration in RPTEC/TERT1 cells. (**A**) Epithelial markers CDH1, occludin, and Keratin-7 and -18 were analyzed by western blot. (**B** and **E**) Cell-to-substrate adhesion as assessed by the ability of RPTEC/TERT1 cells to bind to tissue culture substrate. *N* (number of experiments) = 4, *n* (number of replicates) = 42. Statistical significance was determined using Mann-Whitney test, rank comparison (*P* < 0.01) for Ctrl shRNA vs *CLCN5* shRNA comparison and non-parametric one-way ANOVA (Kruskal–Wallis test) followed by Dunn’s multiple comparison post hoc test for WT vs mutant ClC-5 comparisons. (**C** and **F**) Cell proliferation was measured by staining cells with CFSE and quantifying the fluorescence of the cells at the onset of the experiment and 4 days later. Statistical significance was analyzed using an unpaired *t* test for Ctrl shRNA vs *CLCN5* shRNA comparison, and one-way ANOVA followed by Fisher’s LSD test for WT vs mutant ClC-5 comparisons. (**D** and **G**) collective cell migration, which depends on the integrity of cell–cell contacts, was determined with the wound healing migration assay as indicated in methods. Statistical significance was determined using an unpaired *t* test with equal SD (two-tail) for Ctrl vs *CLCN5* shRNA (*N* = 4) comparison and a one-way ANOVA followed by Tukey’s multiple comparisons *post hoc* test for rClC-5 WT vs mutant (*N* ≥ 3) comparisons. For all experiments, dots represent individual values and columns indicate the mean ± SEM. ^*^, *P* < 0.05; ^*^^*^, *P* < 0.01.

We next explored whether the changes observed in the gene expression profiles by *CLCN5* silencing or loss-of-function ClC-5 mutations correlated with changes in the epithelial characteristics (e.g. substrate adhesion or cell migration). For this purpose, we analyzed epithelial markers’ levels (e.g. CDH1, occludin, and Keratin-7 and -18), cell proliferation, substrate adhesion and collective cell migration. Results in Figure 7A show that *CLCN5* silencing strongly reduced E-Cadherin and Keratin-7 levels, while it had no effect on occludin and Keratin-18 levels. Re-introduction of rClC-5 WT, but not ClC-5 mutants p.(Val523del), p.(Glu527Asp) or p.(Ile524Lys), totally rescued E-cadherin and keratin-7 expression (Fig. 7A). Our results also show that, in comparison with control cells, *CLCN5* silencing increased cell-to-substrate adhesion (*P* < 0.0001) (Fig. 7B) and cell proliferation (*P* = 0.0193) (Fig. 7C), and reduced collective cell migration (*P* = 0.0005) (Fig. 7D). On the other hand, all three ClC-5 mutants increased cell-to-substrate adhesion when compared with the respective control rClC-5 WT (*P* < 0.0001 for p.(Val523del) and p.(Glu527Asp) vs rClC-5 WT and *P* = 0.0018 for p.(Ile524Lys) vs rClC-5 WT) (Fig. 7E). In addition, ClC-5 mutations p.(Val523del) and p.(Glu527Asp) increased cell proliferation (*P* = 0.025 and *P* = 0.0277 vs rClC-5 WT, respectively), while ClC-5 mutation p.(Ile524Lys) did not (*P* = 0.8762 vs rClC-5 WT) (Fig. 7F). Finally, p.(Glu527Asp) (*P* = 0.0104 vs WT) and p.(Ile524Lys) (*P* = 0.0073 vs rClC-5 WT), but not p.(Val523del) (*P* = 0.9254 vs rClC-5 WT), induced a reduction in collective cell migration (Fig. 7G). Taken together, these results suggest that both *CLCN5* silencing and expression of ClC-5 mutants could lead cells into a dedifferentiated state, in agreement with some of the enriched GO BPs found in the previous section, including renal system development and tissue remodeling and morphogenesis.

## Discussion

Besides reports describing the effects of ClC-5 mutations on chloride currents and endosomal acidification, there is a paucity of studies addressing the impact of these mutations on the phenotype and expression profile of PTCs. Consequently, little is known about the putative mechanisms involved in proximal tubule dysfunction secondary to the loss of ClC-5. Moreover, evidences indicate that ClC-5 mutations causing defective proximal tubular endocytosis and endosomal trafficking may not necessarily impair endosomal acidification, suggesting that these processes may not be coupled and that tubular dysfunction in DD1 may not result from reduced endosomal acidification (19,32). In this sense, one of the most striking conclusions of this study is that despite the fact that lack of ClC-5 or the presence of ClC-5 mutations p.(Val523del), p.(Glu527Asp) and p.(Ile524Lys) compromised the endocytic capacity of RPTEC/TERT1 in a similar manner, they do not exert equivalent effects on the gene expression profile of these cells, and only five genes were commonly modulated by these mutations. This suggests that mutant forms of ClC-5 may alter different cellular processes beyond the endocytic pathway. Accordingly, in this work we aimed to discover the mechanisms that underlie tubule dysfunction in DD1 by characterizing the phenotypical consequences of ClC-5 mutations on PTCs.

For this purpose, we have generated novel DD1 cell models by stably transfecting *CLCN5* shRNA and pathogenic ClC-5 mutations into the RPTEC/TERT1 cell line, which maintains many differentiation hallmarks (23). The use of established and well characterized cell lines takes advantage of a uniform genetic background thus avoiding compensatory effects due to currently unknown polymorphisms or mutations in other genes that might be present in other primary cell models. In addition, use of cell models is of special interest in DD1, where renal biopsy is not routinely indicated since (i) laboratory findings and genetic testing can be sufficient for diagnosis and (ii) most DD1 patients are children and it is not ethical to apply an invasive procedure if it cannot provide relevant information when only performed at one specific time point. In this study, we have characterized the ClC-5 mutant proteins and the functionally of the cell models that we have produced, to further identify genes and BPs specifically regulated by *CLCN5* silencing or mutant forms of ClC-5. We chose to study these mutations because they are closely located within the ClC-5 P helix, which is involved in dimer interface formation, and exhibit lack of ClC-5 activity; albeit that, they differentially affect ClC-5 functionality (19,25,26). p.(Ile524Lys) is a class I mutation and abolished currents have been related to its retention in the ER (19). p.(Glu527Asp) is a type 2 ClC-5 mutant, and it lacks currents despite its normal presence in the endosome compartment and partially (30%) reaching the plasma membrane when transfected in HEK-MSR cells (19). Expression of both p.(Glu527Asp) and p.(Ile524Lys) mutants in HEK-293 cells also resulted in impaired endosomal acidification and altered protein stability (19). p.(Val523del) effects on subcellular localization and endosomal acidification have not yet been described. In this regard, we found that RPTEC/TERT1 cells lacking *CLCN5* showed a marked loss of epithelial markers, increased cell to substrate adhesion, reduced collective cell migration and a trend suggesting an increase in cell proliferation, all of them characteristics of epithelial dedifferentiation. This correlated with an altered expression of genes related to ROS, cell–cell adhesion, cell migration, extracellular matrix organization or cell motility among others. In the same direction, Gally *et al*. (33) found that PTCs taken from *CLCN5* KO mice had increased expression of proliferation markers and oxidative scavengers, suggesting that PT dysfunction in *CLCN5* KO mice was associated with oxidative stress, dedifferentiation and increased cell proliferation, which are in agreement with the results found in this study. Moreover, the urinary proteome of patients with Dent disease has been shown to be enriched with proteins actively participating in interstitial matrix remodeling (34). Thereby, our results are aligned with the hypothesis raised by Devuyst and Luciani (20) explaining the potential mechanism by which the loss of ClC-5 may cause proximal tubule dysfunction. According to the authors, and in addition to the impaired trafficking and recycling of apical receptors and the defective receptor-mediated endocytosis, loss of ClC-5 would be also associated with altered lysosomal function. This might compromise the lysosomal mediated-degradation and clearance of autophagosomes containing ubiquitinated proteins and dysfunctional mitochondria, leading to excessive production of ROS. The increase in ROS might alter the integrity of the junctional complex proteins, releasing transcription factors, which will translocate to the nucleus and promote proliferation. It is noteworthy that we did not detect a significant enrichment of GO terms related to phagocytosis in neither *CLCN5*-silenced cells nor cells carrying p.(Val523del), p.(Glu527Asp) nor p.(Ile524Lys) ClC-5 mutations, thereby suggesting the existence of other molecular mechanisms converging on PTCs dedifferentiation and dysfunction. In this sense, and in addition to the abovementioned GO terms, *CLCN5* silencing also altered the expression of genes related to BPs such as anion homeostasis, chemotaxis or response to growth factor. Moreover, terms such as morphogenesis of an epithelial bud and nephron development point at a role of ClC-5 in kidney development. It is worth mentioning that GO terms found in *CLCN5*-silenced RPTEC/TERT1 cells including organ development, ion transport, response to external stimulus, response to wounding, regulation of cell differentiation, chemotaxis and taxis were also found in the gene microarray analysis from proximal S1 and S2 tubules of *CLCN5* KO mice kidneys of the Guggino group (35), indicating that our human cell model mimics the PTCs of the mouse model. By contrast, we did not found terms related to lipid metabolism, which was the class with the greatest number of changes in gene transcript level in the *CLCN5* KO mice (35). That result was surprising because overall changes in lipids have not been reported in Dent disease, likely suggesting species-specific related processes between mouse and human DD1 models. When we analyzed the list of genes altered by *CLCN5* silencing, we found that among the most down-regulated genes there were genes that could be relevant to understand the pathophysiology of DD1, at the proximal tubule level. Such an examples are secretory leukocyte peptidase inhibitor (*SLPI*; logFC -5.01), which has been related to PTCs regeneration (36), *MUC1* (Mucin1; logFC -4.72), whose mutation causes a rare form of tubulointerstitial fibrosis (37), *SLC34A2* (sodium-dependent phosphate transport protein 2B; logFC –4.01), which may contribute to the diminution in the uptake of both sodium and phosphate in the proximal tubules in Dent disease patients, the Rab GTPase *RAB27B* (logFC –1.50), which is involved in exosome secretion, *COL4A4* (Collagen Type IV Alpha 4 Chain; logFC –0.92), which is mutated in patients with Alport syndrome, the kidney-specific Cadherin *CDH16* (logFC –2.25), which is involved in cell–cell adhesions or *KLF4* (Kruppel-like factor 4; logFC –0.99), which has been identified as a renal linage master regulatory transcription factor (38). Taken together, these results suggest that lack of ClC-5 widely affects the phenotype of RPTEC/TERT1 cells, but it remains to be known whether lack of ClC-5 impacts on these processes through its effect on endosomal acidification, altered chloride transport, protein endocytosis, or its participation in macromolecular complexes. For instance, endocytic trafficking contributes to cell adhesion and migration in different ways (39). First, internalization of chemokines by scavenger receptors is essential for sensing the chemotactic gradients, whereas endocytosis and subsequent recycling of chemokine receptors is key for sustaining the responsiveness of migrating cells. Second, endosomal pathways modulate adhesion by delivering integrins to their site of action and supplying factors for focal adhesion disassembly. Finally, endosomal transport also contributes to cell migration by delivering membrane type 1 matrix metalloprotease to the leading edge facilitating proteolysis-dependent chemotaxis.

One of the most striking results of the present work is the reduced number of BPs commonly altered by *CLCN5* silencing and re-introduction of ClC-5 mutations, and also between them, even though all conditions impaired albumin endocytosis and cell differentiation compared to control cells. Only the GO terms ‘extracellular matrix organization’ and ‘extracellular structure organization’ were shared between *CLCN5* silencing and p.(Val523del) and p.(Glu527Asp) conditions. Moreover, although up to 154 genes were modulated by both p.(Val523del) and p.(Glu527Asp), only the GO terms ‘urogenital system development’ and ‘renal system development’ were jointly enriched in these conditions. Interestingly, the BP ‘nephron development,’ which is a child term of the just mentioned GO terms, appeared significantly overrepresented in the *CLCN5* silencing condition. This lack of complete functional equivalence between absence of ClC-5 and presence of ClC-5 mutants in relation to the affected BPs, points to a gain of functionality of the mutated forms of ClC-5. Thus, we hypothesize that, beyond endocytosis impairment, mutant proteins still exhibit a certain degree of functionality that is fully lost in *CLCN5*-silenced cells. In this direction, each of the studied mutations might modify ClC-5 function in distinct ways, yielding to a different gene expression profile in the corresponding cells. That is despite the close location of the mutated residues in ClC-5’s helix P and the fact that amino acids Val523 and Glu527 are highly conserved in all known ClCs (19,25,26).

Interestingly, p.(Val523del) was the condition, among the different mutants studied, with the largest differences in gene profile when compared to the WT form. This could explain, in part, that p.(Val523del) ClC-5 mutation has been found in a pediatric patient with a severe clinical phenotype (40), although the lack of more individuals carrying the same mutation makes impossible to establish such a correlation. Genes altered by p.(Val523del) ClC-5 mutation were mainly involved in cell cycle and proliferation, which are processes that have been linked to a dedifferentiation state, but also in carboxylic acid and anion transport, and renal system development BPs. In this sense, cells carrying p.(Val523del) ClC-5 showed a non-significant increase in cell proliferation compared to rClC-5 WT cells, thereby suggesting that the p.(Val523del)-modulated genes included in proliferation GO terms could indeed be mediating dedifferentiation. On the other hand, we found altered an elevated number of genes from the solute carrier (SLC) group of membrane transport proteins. SLC transporters show high expression levels in metabolically active organs such as the kidney, liver or brain (41), and the kidney has been identified as one of the target organs for most high expression of SLCs-mediated diseases (42). For instance, we found the type I sodium-dependent phosphate transporters *SLC17A1* (NPT1) and *SLC17A3* (NPT4), as the two most up-regulated genes in this condition, as well as *SLC27A2* (Fatty Acid Transporter FATP2), *SLC16A4* (Monocarboxylate Transporter 4 MCT4) and *SLC4A4* (Sodium Bicarbonate Cotransporter NBC1), all of them being involved in renal diseases (42). To cite some of them related to proximal tubule dysfunction, *SLC17A1* mutations cause dysregulation of urate handling by the proximal tubule cells (43) and *SLC4A4* mutations cause defective reabsorption of HCO_3_^−^, leading to proximal tubule acidosis (44). By contrast, neither *CLCN5*-silenced cells nor any mutant condition showed an altered expression of the sodium-bile acid cotransporter *SLC10A2*, which was one of the gene transcripts most increased in transcript number (17 fold) in the C*LCN5* knockout mice proximal tubules of the Guggino group (35). However, we found that p.(Val523del) cells had a significant enrichment of the BP ‘bile acid synthesis and transport,’, and genes contained in this GO term, such as the bile acid receptor *NR1H4*, and the nuclear receptor NR1D1 or the very long-chain acyl-CoA synthetase *SLC27A2*, were also among the most up-regulated genes in p.(Val523del) cells. Thus, the fact that p.(Val523del) ClC-5 up-regulates so many genes codifying for apical and basolateral membrane co-transporters may suggest the existence of compensatory pathways to overcome the defective receptor-mediated endocytosis caused by ClC-5 loss-of-function. Besides that, and as mentioned above, renal development was another BP altered in p.(Val523del) cells. Amid the genes belonging to this BP, we found highly up-regulated (logFC 2.31) the transcription factor *HES1* (hairy and enhancer of split-1), previously identified as a renal linage master regulatory transcription factor, playing an important role in the Notch signaling pathway (38). As for p.(Val523del) down-regulated genes, it is remarkable to note that some of the top down-regulated genes, such as *CDH1*, *MFAP5* or *LUM*, participate in extracellular matrix organization and cell–cell adhesion. This effect could in part explain the increased cell-to-substrate adhesion of the cells carrying the p.(Val523del) mutation. Finally, it is also worth mentioning that cells carrying p.(Val523del) mutation downregulate *SLC3A1* gene, which codifies for the amino acid transporter ATR1 that is mutated in patients affected of cystinuria (42).

As mentioned earlier, Glu527 is one of the most conserved amino acids and is present in all the known ClCs, including those from plants, yeast, *Escherichia coli*, cyanobacteria, fish and mammals (19,26). In addition, it has been previously described that the p.(Glu527Asp) ClC-5 mutant has a dominant negative effect on endosomal acidification (19), and mutation of the corresponding residue in ClC-0 results in a reversion of voltage dependence, i.e. currents were activated by hyperpolarization instead of depolarization (45). It is striking that, in our cell model, a large part of the BPs altered by the p.(Glu527Asp) mutation were related to tissue remodeling, morphogenesis, differentiation and development. Interestingly, the BPs ‘Organ development’ and ‘organ morphogenesis’ were the second and eighth GO terms, respectively, with the greatest number of significantly changed transcripts in the *CLCN5* KO mice of the Guggino group (35). Other BPs, such as sialic acid transport, sodium ion transmembrane transport and, cell substrate adhesion, BMP signaling pathway or T cell activation appeared altered in RPTEC/TERT1 cells carrying p.(Glu527Asp) ClC-5. In this sense, genes related to T cell activation, such as genes of the human leukocyte antigen (HLA) system (*HLA-DRB1*, logFC 1.79; *HLA-DMA*, logFC 1.55; and *HLA-DPA1*, logFC 1.44) or the lymphocyte cytosolic protein 1 (*LCP-1*, logFC -1.46) and Thy-1 cell surface antigen (*THY1*, logFC -1.44) are among the most up or down regulated genes by p.(Glu527Asp) re-introduction. These results are consistent with microarray data obtained from intestine of *CLCN5* KO mice showing altered expression of genes implicated in the immune system (46), and with the proposed role for ClC-5 in the immunopathogenesis of ulcerative colitis (47). In addition, we also found biological pathways related to bone remodeling. In this sense, it is well known that the kidney is the major organ involved in the regulation of calcium and phosphate homeostasis, which is essential for bone mineralization and development (48). Thus, an enrichment of such BPs could be indeed reflecting an altered phosphate regulation in PTCs. Moreover, alteration of these processes in PTCs could be related to the increased bone turnover previously described in the *CLCN5* KO mouse model of Dent’s disease, likely explaining the propension to altered bone homeostasis in young Dent’s patients (49).

Curiously, introduction of the ClC-5 mutant p.(Ile524Lys) in RPTEC/TERT1 cells only altered the expression of a reduced number of genes, yielding, among the different mutants studied, the gene expression profile that more closely resembled that of the rClC-5 WT condition. These results were unexpected considering that (i) the p.(Ile524Lys) mutant presented the highest degree of ER localization among the different ClC-5 mutants studied, and (ii) the lack of a correspondence between p.(Ile524Lys) mRNA and protein levels might indicate that this mutant exhibits reduced protein stability or impaired post-translational processing. The increased ER retention of p.(Ile524Lys), however, does not translate to an induction of the UPR, suggesting that p.(Ile524Lys) would be rapidly targeted to proteasomal degradation without displaying and ER stress gene signature. Proteasomal degradation, in turn, could explain the reduced levels of p.(Ile524Lys) protein that we detected by western blot. A possible explanation for the reduced phenotypic effects of p.(Ile524Lys) would be that, although most of the proteins would be retained in the ER, a small but sufficient amount of the p.(Ile524Lys) proteins would manage to escape from the ER and reach its functional localization. This hypothesis would imply that p.(Ile524Lys) is, beyond its retention in the ER, a functional protein able to produce chloride currents. The reduced number of genes modulated by p.(Ile524Lys) did not allow to find statistically enriched GO terms. However, an analysis of the DEGs in p.(Ile524Lys) cells shed some light about the potential processes altered by this mutation. In this sense, the top-down regulated genes, *CDH1* (E-cadherin) and *KRT7* (keratin-7), correspond to well-established epithelial markers. This expression profile is aligned with the reduced collective cell migration observed in rClC-5 p.(Ile524Lys) cells. Moreover, the third most down-regulated gene, *CATSPER1*, corresponds to a voltage-gated calcium channel, and the fifth, *SLC38A8*, to a putative sodium-dependent amino-acid/proton antiporter. On the other hand, *PREX1*, a guanine nucleotide exchange factor for RAC1, and the ferroxidase enzyme Ceruloplasmin (CP) were the most up-regulated genes by p.(Ile524Lys). *PREX1*, which is one of the few genes altered by all the three ClC-5 mutations studied, has been identified as an important factor in tumor cell invasion and metastasis in a number of cancer models (50). As for *CP*, it has been described that it plays an important role in cellular iron homeostasis and could protect kidney against a damage from iron excess (51). Interestingly, *CP* was also found up-regulated in KO mice of the Guggino group, and the molecular function ‘iron ion binding’ appeared in sixth position in the GO miner analysis of DEGs (35).

In conclusion, in this work we have generated new cell models of Dent disease that accurately reproduce ClC-5 defects previously described in *CLCN5* KO mice and DD1 patients and we have demonstrated that, besides the established critical function of ClC-5 in endocytosis, there are other mutation-associated pathways that could be relevant for the etiopathogenesis of DD1, likely explaining the phenotypic variability of DD1 patients. In this sense, we found that BPs related to kidney development, anion homeostasis, organic acid transport, extracellular matrix organization and cell migration, were among the pathways that more likely could explain the pathophysiology of DD1.

## Materials and Methods

### Cell culture

Renal proximal tubule epithelial cells RPTEC/TERT1 were obtained from the American Type Culture Collection (#CRL-4031, ATCC®). RPTEC/TERT1 were cultured in Dulbecco’s Modified Eagle Medium: Nutrient Mixture F-12 (1:1, v/v) (#31331093, Thermo Fisher Scientific) supplemented with 20 mM HEPES (#15630–080, Gibco), 60 nM sodium selenite (#S9133, Sigma-Aldrich), 5 μg/ml transferring (#T1428, Sigma-Aldrich), 50 nM dexamethasone (#D8893, Sigma-Aldrich), 100 U/ml penicillin and 100 μg/ml streptomycin (#15240–062, Gibco), 2% fetal bovine serum (#10270, Gibco), 5 μg/ml insulin (#I9278, Sigma-Aldrich), 10 ng/ml epidermal growth factor (#E4127, Sigma-Aldrich) and 3 nM triiodothyronine (#T5516, Sigma-Aldrich). Cultures were maintained at 37°C in a 5% CO_2_ atmosphere. Unless otherwise indicated, cells were cultured for 10 days to allow cell differentiation. Cells were routinely tested for mycoplasma contamination.

### Gene silencing

For *CLCN5* silencing, the MISSION® TRC shRNA transfer vector containing the ClC-5 shRNA target sequence CACCGAGAGATTACCAATAA (#TRCN0000043904, Sigma-Aldrich) was co-transfected with the third generation vectors VSVG, RTR2 and PKGPIR, which provide the envelope, packaging and reverse-expressing proteins, respectively, into HEK-293T cells. Supernatants containing viral particles were then harvested, supplemented with 10% FBS, 1% non-essential amino acids and 8 μg/mL polybrene (#TR-1003, Sigma-Aldrich) and added to RPTEC/TERT1 cells, followed by antibiotic-mediated selection (8 μg/mL puromycin, #ant-pr, Invivogen).

### Vectors and site-directed mutagenesis

For shRNA rescue experiments, the canonical form (NM_000084.4; mRNA variant 3) of WT human *CLCN5* was cloned into pDONR vectors (pDONR™221, #12536–017, Invitrogen) using the Gateway cloning system (Invitrogen). In order to escape from degradation by the RISC complex, silent mutations (c.[99C > T; 100C > A; 102A > G; 105G > A; 108T > C; 111C > A; 114T > C]) were introduced in the shRNA targeting sequence of human *CLCN5*. Over the shRNA-rescuing ClC-5 vector, we introduced the following mutations in the *CLCN5* gene: p.(Val523del) (c.1566-1568del), p.(Glu527Asp) (c.1581A > T) and p.(Ile524Lys) (c.1571T > A). In addition, a HA-tag was also added in the C-terminus of each cDNA. Subsequently, all inserts were sub-cloned to an expression vector containing hygromycin resistance (pLenti CMV Hygro DEST 117–1, Addgene) using the Gateway recombination system. All constructs generated were stably transduced into previously *CLCN5-*silenced cells using lentiviral particles produced in HEK-293T cells and were subsequently selected with 400 μg/ml hygromycin (#ant-hg-5, Invivogen). Site-directed mutagenesis was performed with the QuikChange® II XL Site-Directed Mutagenesis Kit (#200522, Agilent Technologies) and primers were designed using the QuikChange Primer Design tool (Agilent Technologies).

### RNA extraction and qPCR

Total RNA was isolated from cells using TRIzol® Reagent (#15596–026, Life Technologies) following the manufacturer’s protocol, and 1000 ng of RNA in a final volume of 20 μl was retro-transcribed using the High-Capacity cDNA Reverse Transcription Kit (#4387406, Applied Biosystems). For each probe to analyze, 20 ng of cDNA was amplified in a 10 μl final volume of reaction mix containing SYBR Green MasterMix (#A25742, Applied Biosystems) or TaqMan MasterMix (#4369016, Applied Biosystems), according to the manufacturer’s instructions. Endogenous levels of *CLCN5* mRNA were measured using specific probes targeting the intact shRNA target sequence (Endogenous *CLCN5* primers: 5′-GGGATAGGCACCGAGAGAT-3′ and 5′-GGTTAAACCAGAATCCCCCTGT-3′). Levels of re-introduced (exogenous) forms of *CLCN5* were measured using specific probes against the C-terminal HA-tag, which is only present in re-introduced forms (Exogenous *CLCN5* primers: 5′-GGTTACACACAACGGGCGAT-3′, and 5′-CGTAATCTGGAACATCGTA-3′). Total levels of *CLCN5* were measured with a commercial TaqMan probe (#Hs00163986_m1, Applied Biosystems). Data were normalized against TBP using the following primers: 5′-CGGCTGTTTAACTTCGCTTC-3′ and 5′-CAGACGCCAAGAAACAGTGA-3′, or the commercial TaqMan probe *TBP* Hs00427620_m1 (Applied Biosystems). In order to validate the microarray analysis, the following TaqMan probes was used: (Applied Biosystems): *STEAP1* (Hs00185180_m1), *ZPLD1* (Hs00604192_m1), *PTPRD* (Hs00369913_m1), *CDH1* (Hs01023895_m1), EMX2 (Hs00244574_m1), *NR1H4* (Hs01026590_m1), and *EHF* (Hs00171917_m1). Analysis was performed using the 7900HT Sequence Detection System (Applied Biosystems). Relative expression fold change was determined by the comparative 2^(−ΔΔCT)^ method after normalizing to TBP. For the analysis of XBP-1 splicing, we used the following primers: 5′-AAACAGAGTAGCAGCGCAGACTGC-3′ and 5′-TCCTTCTGGGTAGACCTCTGGGAG-3′.

### Western blot

Cells were lysed in SET buffer (10 mM Tris–HCl pH 7.4, 150 mM NaCl, 1 mM EDTA and 1% SDS) and the protein concentration was quantified by the BCA assay (#23225, Thermo Fisher Scientific). Equal amounts of whole cell extracts (ranging from 20 to 80 μg of protein depending on the antibody) were resolved in 10% SDS-PAGE gels and transferred to PVDF membranes (#ISEQ00010, Millipore). Membranes were blocked with 5% non-fat dry milk diluted in PBS-T (1x PBS, 0.1% Tween-20) for 1 h and incubated overnight at 4°C with the appropriated antibodies: HA (1:1000 dilution, #11867423001, Roche), β-tubulin (1:5000 dilution, #T4026, Sigma-Aldrich), E-Cadherin (1:1000 dilution, #610181, BD Transduction Labs), Occludin (1:1000 dilution, #71–1500, Invitrogen), PERK (1:1000 dilution, #5683 T, Cell Signaling), Cytokeratin-7 (undiluted, #790–4462, Ventana Medical Systems) and Cytokeratin-18 (1:1000 dilution, #51582, Santa Cruz). Membranes were then incubated with the corresponding secondary antibodies (rabbit anti-mouse IgG/HRP, #P0260, Dako, and goat anti-rat IgG/HRP, #A9037, Sigma-Aldrich) at a 1:5000 dilution. Membranes were visualized using chemiluminescence reagent (#WBLUF0500, Millipore) and exposed on Odyssey Fc Imaging System (Li-Cor).

### Immunocytochemistry

RPTEC/TERT1 cells were cultured on glass coverslips (#0111450, Marlenfeld GmbH & Co. KG) for 10 days. Cells were then washed in cold PBS and fixed in −20°C methanol for 5 min at room temperature. Aldehyde groups were quenched in 50 mM NH_4_Cl/PBS for 30 min and non-specific binding sites were blocked with 5% BSA in PBS for 60 min. Coverslips were incubated overnight at 4°C with a 1:100 dilution with one of the following primary antibodies: HA (#867423001, Roche), KDEL (#Ab10C3, Abcam), Rab5 (#3547S, Cell Signaling) and N-Cadherin (#610920, BD Transduction Labs), followed by incubation with the corresponding fluorescent-conjugated secondary antibodies (1:500 dilution, #A11004, #A28175, #A11011, #A27034, #A21247, and #A27012, Thermo Fisher Scientific) for 1 h at room temperature. Finally, cells were incubated with Hoechst 33342 (1:2000 dilution, #H1399, Invitrogen) for 5 min to stain cell nuclei. Coverslips were then mounted on slides with Prolong diamond mounting medium (#P36961, Thermo Fisher Scientific) and fluorescence labeling was visualized in a confocal spectral Zeiss LSM 980 microscope. Acquired images were processed using ImageJ software (52), and the co-localization analysis was performed using the ImageJ JACoP plugin (53). Co-localization was measured using Pearson’s and Manders co-localization coefficients.

### Glycosylation assay

RPTEC/TERT1 cells were lysed using RIPA buffer supplemented with protease inhibitor cocktail (#P8340, Sigma-Aldrich) and equal amounts of whole cell extracts were digested for 18 h with endoglycosidase H (Endo H, #P0702S, New England Biolabs) or peptide N-glycosidase F (PNGase F, #P0704S, New England Biolabs) enzymes following the manufacturer’s instructions.

### Albumin uptake

Albumin uptake was measured to investigate receptor-mediated endocytosis. RPTEC/TERT1 cells were seeded on glass coverslips (Marlenfeld GmbH & Co. KG) and grown for 10 days. To measure albumin uptake, cells were exposed to 50 μg/mL Alexa Fluor 488-conjugated Albumin (#A13100, Thermo Fisher Scientific) for 60 min. At the end of the incubation period, cells were washed six times with ice-cold PBS and fixed in −20°C methanol for 5 min. Instead of stripping the cells, non-specifically bound albumin was excluded by using the confocal orthogonal view. To reveal cellular perimeter, slides were incubated 1 h at room temperature with a 1:5000 dilution of Phalloidin conjugated to tetramethyl-rhodamine-isothiocyanate (TRITC, excitation/emission: 540–545/570–573 nm; Sigma-Aldrich). Cell nuclei were stained with Hoechst 33342 (1:2000 dilution, #H1399, Invitrogen) for 5 min at room temperature and slides were mounted with Prolong diamond mounting medium (#P36961, Thermo Fisher Scientific). Images were acquired with a 63X objective on a confocal laser scanning microscope (Zeiss LSM 980) and processed using ImageJ software. Co-localization analysis was performed using the ImageJ JACoP plugin.

### DNA microarray

Total RNA for DNA microarray was isolated as indicated before. RNA quality was checked using Bioanalyzer nano assay (Agilent Technologies). RNA samples representing five separate experiments from each of the conditions were used. Ten independent microarrays were performed using the Clariom D arrays (#902922, Affymetrix-Genechip array) according the manufacturer’s protocol. Briefly, 200 ng of total RNA was retro-transcribed using WT pico HT Kit (#902622, Thermo Fisher Scientific). The resulting cDNA was fragmented, labeled and hybridized using the GeneAtlas hybridization, wash, and stain kit for WT array strips (#901667, Thermo Fisher), and finally, the Clariom D array was placed in the GeneTitan MC System for scanning (#00–0373, Affymetrix).

### Over representation analysis

GO terms over-representation analysis was performed using the webserver g:Profiler (https://biit.cs.ut.ee/gprofiler/gost) as described in (54).

### XBP1 splicing assay

XBP-1 mRNA splicing was analyzed by PCR in untreated cells or control cells treated with ER stress inducers Brefeldin-A (1 μM, #B6542, Sigma-Aldrich) and Tunicamycin (5 μM, #T7765, Sigma-Aldrich). RNA was then isolated and transcribed to cDNA as described previously. For each condition, one μl of cDNA was amplified in a 25 μl final volume of reaction mix containing 2.5 mM MgCl_2_, 0.2 mM dNTPs, 0.2 μM forward primer (5′-AAACAGAGTAGCAGCGCAGACTGC-3′), 0.2 μM reverse primer (5′-TCCTTCTGGGTAGACCTCTGGGAG-3′), and 1 unit of Taq polymerase (Bioline, Meridian Bioscience). PCR conditions were as follow: an initial melting step of 94°C for 2 min; then 30 cycles of 94°C for 30 s, an annealing step of 60°C for 30 s, and 72°C for 30 s; followed by a final elongation step of 72°C for 5 min. Cyclophilin A was used as a housekeeping control gene (forward primer 5′-ATGGTCAACCCCACCGTGTTC-3′; reverse primer 5′-TTCGAGTTGTCCACAGTCAGCAAT-3′). PCR products were separated in 3% agarose gels.

### Cell proliferation

Cell proliferation was performed as previously described (55). Briefly, cells were incubated with 5 μM carboxyfluorescein succinimidyl ester (CFSE, #21888, Sigma-Aldrich) for 10 min at 37°C. The unbound CFSE was quenched by washing cells twice in complete medium. An aliquot of cells was used to measure cell fluorescence at the onset of the experiment. The rest of labeled cells were seeded on tissue plates and incubated at 37°C until reaching a confluency of 40–60% (3–4 days). At the end of this period, fluorescence of daughter cells was measured. Cell fluorescence was measured on a FACS calibur flow cytometer (Becton Dickinson) and proliferation indices were determined using the Cell Quest software (Becton Dickenson).

### Cell adhesion

RPTEC/TERT1 cells cultured for 10 days were trypsinized, washed twice with culture medium to eliminate trypsin and counted. Fifty thousand cells/well were then seeded onto two duplicated 96-well plates for 60 min at 37°C. After this period, unattached cells from one of the plates were removed by washing cells twice with PBS, followed by an additional incubation period of 60 min in medium to facilitate cell recovery. The amount of attached cells (from the washed plate) and the total cells (from the unwashed plate) was determined using XTT assay (# 11465015001, Sigma-Aldrich) following the manufacturer’s instructions.

### Wound healing migration assay

For wound healing migration assay (also termed scratch assay), 2.25 × 10^4^ cells were seeded on each of the two compartments of silicone culture inserts (#81176, Ibidi) and grown for 10 days. At the onset of the experiment, the silicone culture insert was removed leaving a defined 500 μm (according to the manufacturer’s information) cell-free gap between the two compartments previously seeded with cells. Cells were then gently washed twice with medium to remove cell debris, and gap closure (wound healing) was monitored by obtaining digital images of the cell-covered area at the onset of the experiments and 21 hours later with a Thunder microscope (Leica). Area measurements at each time point were performed using ImageJ software.

### Statistical analysis

All data sets were first tested for normality using the D’Agostino and Pearson omnibus normality test. Statistical significance was determined by Student’s *t* test (continuous data, two groups) or one-way analysis of variance (ANOVA) (for the comparison of more than two groups) followed by Turkey’s *post hoc* test. If data were not normally distributed, we used Mann–Whitney’s unpaired *t* test (two groups) or the non-parametric ANOVA (Kruskal–Wallis) (more than two groups) followed by Dunn’s *post hoc* test. All statistical analyses were performed using GraphPad Prism 6 software (RRID:SCR_002798). Values are expressed as mean ± SEM. Criteria for a significant statistically significant difference were: ^*^P < 0.05; ^*^^*^P < 0.01. Each specific test is indicated in figure legends.

## Supplementary Material

Supplemental_Data_Duran_et_al_revised_ddab131
