# Supplementary material for: Novel Dent disease 1 cellular models reveal biological processes underlying ClC-5 loss-of-function
Source: Hum Mol Genet. 2021 May 13;30(15):1413–28. doi: 10.1093/hmg/ddab131 (PMC8283206; doi:10.1093/hmg/ddab131)
Supplement: Supplemental_Data_Duran_et_al_revised_ddab131 [file supplemental_data_duran_et_al_revised_ddab131.docx]

**Supplemental Data**


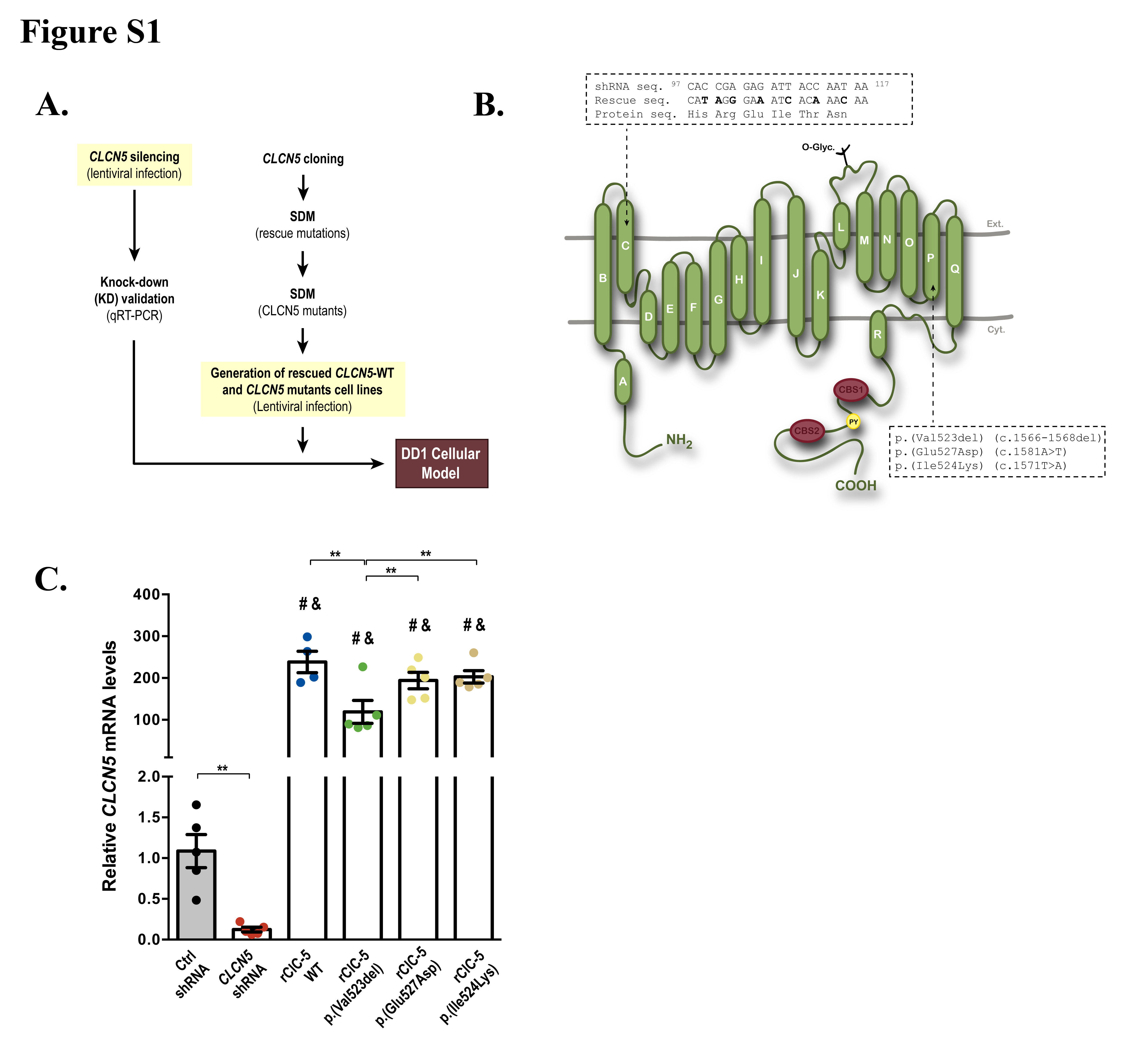
**Figure S1. Generation of Dent disease 1 cell models.** To explore the molecular mechanisms underlying PTCs dysfunction in DD1, we have generated stable RPTEC/TERT1 cell lines silenced for *CLCN5* gene or carrying the pathogenic ClC-5 mutations p.(Val523del), p.(Glu527Asp) or p.(Ile524Lys). (A) *CLCN5* was initially silenced in RPTEC/TERT1 cells using lentiviral shRNA vectors, and cells carrying *CLCN5* silencing were selected with the antibiotic puromycin. To re-introduce wild-type (WT) or mutant ClC-5, we introduced silent mutations in the shRNA target sequence to prevent RISC-mediated degradation. Subsequently, we mutated ClC-5 residues Val523, Glu527 and Ile524 and we transduced the previously ClC-5 silenced cells. Cells carrying both *CLCN5* shRNA and re-introduced ClC-5 forms were isolated using dual antibiotic selection (puromycin and hygromycin). (B) Scheme depicting ClC-5 the canonical 746-amino acid ClC-5 protein with its 18 membrane spanning α-helices, and the localization of shRNA target sequences and mutations p.(Val523del), p.(Glu527Asp) and p.(Ile524Lys) within the helix P of ClC-5. (C) mRNA levels of total (endogenous and exogenous) *CLCN5* from control cells (Ctrl shRNA), *CLCN5*-silenced cells (*CLCN5* shRNA), and *CLCN5*-silenced cells carrying WT (rClC-5 WT) and mutant (rClC-5 p.(Val523del), rClC-5 p.(Glu527Asp) and rClC-5 p.(Ile524Lys)) ClC-5 were measured by RT-qPCR using a commercial TaqMan probe, as indicated in Methods. Statistical significance was determined using an unpaired t test (two-tailed) for Ctrl shRNA vs *CLCN5* shRNA comparison, and one-way ANOVA followed by Tukey’s multiple comparisons test for the rest of comparisons. All values were normalized to Ctrl shRNA levels. ** p < 0.01; # p < 0.01 compared to Ctrl shRNA; & p < 0.01 compared to CLCN5 shRNA.

**
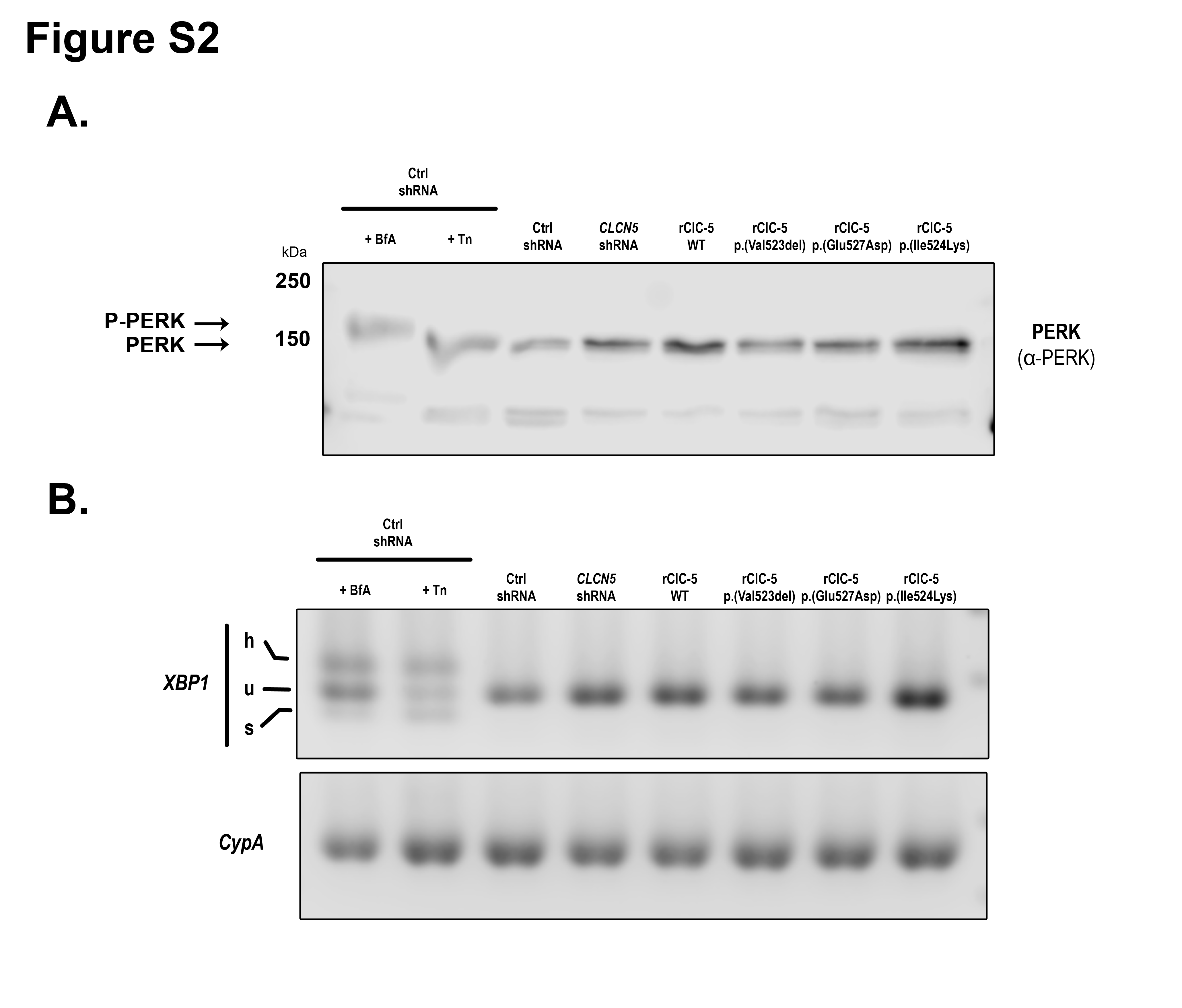
**

**Figure S2. Expression of mutant ClC-5 proteins does not induce ER stress in RPTEC/TERT1 cells.** To investigate whether the expression of ClC-5 mutants could be inducing the Unfolded Protein Response (UPR) and ER stress as a result of their accumulation in the ER, we checked the activation status of markers of the main branches of ER stress, i.e, PERK and IRE1α pathways. As positive controls, cells were treated with the well-established ER stress inducers brefeldin-A (BfA) or Tunicamycin (Tn). (A) Western blots showing that only BfA, but not Tn, *CLCN5* silencing or expression of ClC-5 mutants induced a shift in the molecular weight of PERK, which has been associated with increased phosphorylation and activation of this protein kinase. (B) The activation of the IRE1α pathway was assessed by monitoring the splicing of XBP-1 mRNA in cells expressing WT or mutant ClC-5 (h-hybrid, u-unspliced and s-spliced refer to the forms of XBP-1 mRNA identified in agarose gels). In this case, both BfA and Tn induced XBP-1 cleavage. On the other hand, neither *CLCN5* silencing nor any of the ClC-5 mutants induced detectable cleavage of XBP-1.

**
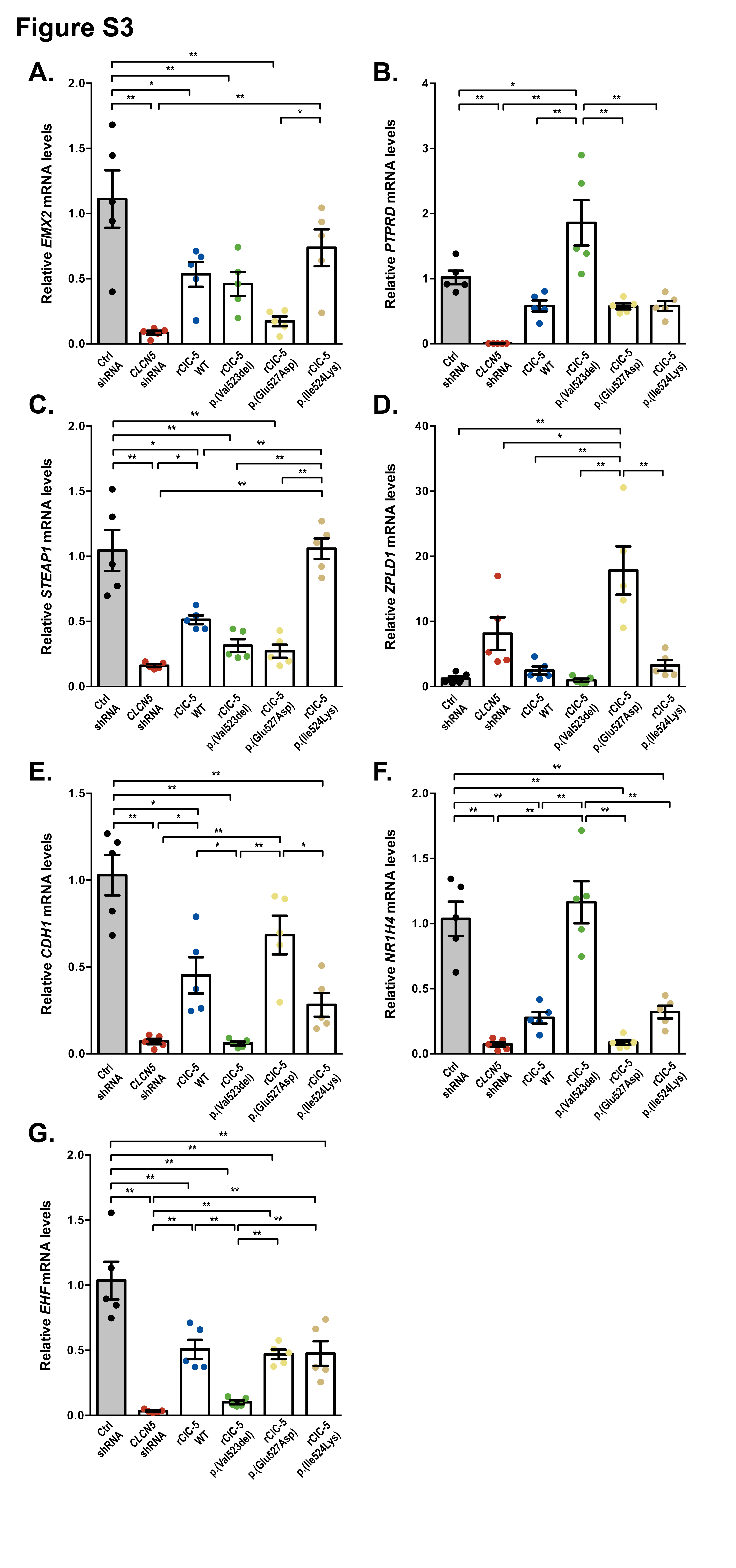
**

**Figure S3. mRNA expression levels of DNA microarray validation genes**. To validate the reliability of the results obtained from the DNA microarray, the expression levels of *EMX2*, *PTPRD*, *STEAP1*, *ZPLD1*, *CDH1*, *NR1H4* and *EHF* genes were analyzed by qRT-PCR. Validation genes were selected among those that meet the following requirements: i) their expression was altered by some of the mutations compared to the WT condition and, ii) its expression was also modified by the silencing of *CLCN5* and totally or partially restored by ClC-5 WT re-introduction. All genes showed an expression pattern that correlated with that observed in the DNA microarray. Dots represent individual values and columns indicate the mean ± SEM, n = 5. Statistical significance was determined using one-way ANOVA followed by Tukey’s multiple comparisons post hoc test. *, p < 0.05; **, p < 0.01.

Supplementary Tables

Table S1. Top 20 down-regulated genes by *CLCN5* silencing

|  | *CLCN5* shRNA vs Ctrl shRNA | | rClC-5 WT vs *CLCN5* shRNA | |
| --- | --- | --- | --- | --- |
| Gene Symbol | logFC | adj.p.val. | logFC | adj.p.val. |
| SLPI | -5,012258677 | 7,25872E-18 | 2,490688757 | 1,48614E-10 |
| PTPRD | -4,774660354 | 5,73261E-18 | 4,076780087 | 4,04796E-16 |
| MUC1 | -4,725636323 | 1,63254E-20 | 2,641888478 | 1,76224E-14 |
| SOSTDC1 | -4,167052862 | 4,70927E-14 | 1,301344707 | 0,000249537 |
| EHF | -4,10574689 | 4,93531E-19 | 3,279045767 | 2,02258E-16 |
| SLC34A2 | -4,012080704 | 3,34897E-16 | 3,063994515 | 2,63198E-13 |
| ARHGEF38 | -3,797136664 | 7,14438E-15 | 0,983246057 | 0,000762179 |
| LIX1 | -3,7193965 | 9,11766E-14 | 0,883886992 | 0,005773676 |
| CDH1 | -3,697591042 | 7,14438E-15 | 2,466205116 | 1,48614E-10 |
| CXCL6 | -3,547100112 | 5,63081E-12 | 2,069829383 | 7,08157E-07 |
| ANPEP | -3,497712417 | 2,57939E-14 | 3,384013567 | 1,46952E-13 |
| C3 | -3,431960618 | 7,14438E-15 | 1,977200085 | 3,2444E-09 |
| PTGFR | -3,339062198 | 4,36867E-13 | 0,641977911 | 0,040806607 |
| CDH3 | -3,31371677 | 5,73261E-18 | 0,515618763 | 0,004751952 |
| LOC101927630 | -3,312773819 | 3,67966E-14 | 1,846828299 | 2,36354E-08 |
| DPP4 | -3,30097477 | 1,0374E-17 | 2,391987241 | 4,87344E-14 |
| MAOA | -3,287033568 | 1,28465E-14 | 1,55734935 | 1,80241E-07 |
| CSGALNACT1 | -3,242946306 | 1,80999E-13 | 0,723010169 | 0,012523965 |
| KRT7 | -3,191449694 | 1,43739E-14 | 1,843985356 | 5,77037E-09 |
| EMP1 | -3,03942192 | 4,08616E-14 | 1,579039068 | 9,30668E-08 |

Table S2. Top 20 up-regulated genes by *CLCN5* silencing

|  | *CLCN5* shRNA vs Ctrl shRNA | | rClC-5 WT vs *CLCN5* shRNA | |
| --- | --- | --- | --- | --- |
| Gene Symbol | logFC | adj.p.val. | logFC | adj.p.val. |
| SLC14A1 | 5,08104134 | 4,79114E-14 | -3,977038387 | 4,18868E-11 |
| TTC29 | 3,679206623 | 1,14704E-12 | -1,067780535 | 0,002680413 |
| TMEM178A | 3,350740312 | 3,79059E-16 | -2,614100467 | 2,12546E-13 |
| ACSM3 | 3,04935039 | 3,89094E-14 | -3,482219987 | 7,56065E-15 |
| DIRAS2 | 3,014635521 | 3,92929E-15 | -1,807712049 | 6,97955E-10 |
| RAB3B | 2,969127618 | 7,25872E-18 | -1,363054288 | 6,97955E-10 |
| LINC01508 | 2,871168794 | 5,11238E-11 | -1,560351427 | 1,15104E-05 |
| ADAMTS16 | 2,856340255 | 2,26768E-10 | -2,292388697 | 6,32122E-08 |
| ITGA11 | 2,820938391 | 1,54507E-14 | -2,796221655 | 5,08676E-14 |
| KIT | 2,784145733 | 5,37046E-11 | -1,970813304 | 1,67632E-07 |
| NTM | 2,766697018 | 4,97022E-13 | -1,45799613 | 5,89012E-07 |
| FAM189A1 | 2,617138339 | 2,24867E-15 | -1,498680451 | 1,08268E-09 |
| SLC16A10 | 2,541950339 | 3,98234E-11 | -1,243742469 | 4,16723E-05 |
| ST8SIA2 | 2,44775401 | 7,32502E-14 | -1,116925328 | 1,39984E-06 |
| PGM5 | 2,44469443 | 1,80999E-13 | -1,476028667 | 2,36354E-08 |
| ZPLD1 | 2,429899703 | 1,65216E-12 | -1,687245584 | 1,20979E-08 |
| MAP3K7CL | 2,357575992 | 2,55733E-11 | -1,568185814 | 2,48828E-07 |
| SFRP4 | 2,331536894 | 7,59206E-14 | -1,353862223 | 2,35953E-08 |
| HMCN1 | 2,26960979 | 1,77002E-10 | -2,34963613 | 4,42059E-10 |
| ZNF385D | 2,161982015 | 1,33712E-12 | -1,526276707 | 6,79872E-09 |

Table S3. Top 20 down-regulated genes by re-introduction of p.(Val523del) ClC-5 mutant

|  | rClC-5 p.(Val523del) vs rClC-5 WT | |
| --- | --- | --- |
| Gene Symbol | logFC | adj.p.val. |
| CDH1 | -2,2699238 | 2,1101E-09 |
| MFAP5 | -2,1515998 | 2,1095E-08 |
| C3 | -2,0811644 | 2,9962E-09 |
| LUM | -1,8057315 | 5,6068E-07 |
| KRT7 | -1,7459962 | 2,9746E-08 |
| OPRPN | -1,6387219 | 1,2899E-05 |
| EHF | -1,5793078 | 3,4844E-09 |
| WNT7A | -1,5521822 | 2,1101E-09 |
| SLCO2B1 | -1,4213937 | 4,7274E-06 |
| ADGRF1 | -1,4152315 | 1,2046E-09 |
| FABP3 | -1,3842962 | 4,9308E-06 |
| MAP1B | -1,3263065 | 8,4725E-07 |
| MUC1 | -1,3139873 | 7,0926E-08 |
| ENTPD1 | -1,3088421 | 8,5295E-06 |
| MACC1 | -1,296171 | 7,5965E-09 |
| LCN2 | -1,2896225 | 0,00088447 |
| TAGLN | -1,2727844 | 1,6752E-05 |
| UCA1 | -1,2380877 | 5,3125E-08 |
| PDE1C | -1,2030441 | 7,0256E-06 |
| ZDHHC15 | -1,1699512 | 3,5396E-05 |

Table S4. Top 20 up-regulated genes by re-introduction of p.(Val523del) ClC-5 mutant

|  | rClC-5 p.(Val523del) vs rClC-5 WT | |
| --- | --- | --- |
| Gene Symbol | logFC | adj.p.val. |
| SLC17A1 | 3,04935497 | 7,574E-09 |
| SLC17A3 | 2,81283694 | 1,4946E-08 |
| NR1H4 | 2,59213551 | 7,3806E-11 |
| CLDN2 | 2,44095847 | 3,5125E-07 |
| NR1D1 | 2,35073551 | 2,1101E-09 |
| HES1 | 2,31656296 | 5,3125E-08 |
| CXCL6 | 2,11838805 | 6,8979E-07 |
| APCDD1L-AS1 | 1,9331687 | 2,7107E-08 |
| RPL22L1 | 1,88554225 | 4,4577E-13 |
| SLC27A2 | 1,81289443 | 2,919E-07 |
| KCNJ15 | 1,77286807 | 0,00010917 |
| EFHB | 1,71213441 | 3,5743E-07 |
| LINC01291 | 1,70389896 | 1,4025E-07 |
| TCF4 | 1,67893034 | 2,8791E-08 |
| KLF10 | 1,6788553 | 1,941E-09 |
| MATN2 | 1,59817147 | 1,2046E-09 |
| PTPRD | 1,54984402 | 4,1356E-07 |
| PRKAR2B | 1,54571457 | 5,1072E-07 |
| ANPEP | 1,51082471 | 2,1612E-06 |
| RELN | 1,49928918 | 2,328E-05 |

Table S5. Top 20 down-regulated genes by re-introduction of p.(Glu527Asp) ClC-5 mutant

|  | rClC-5 p.(Glu527Asp) vs rClC-5 WT | |
| --- | --- | --- |
| Gene Symbol | logFC | adj.p.val. |
| MFAP5 | -2,3750789 | 8,8178E-09 |
| FLG | -2,0583374 | 5,6017E-09 |
| NETO1 | -1,8661723 | 4,6023E-06 |
| EMX2 | -1,5523818 | 7,3078E-08 |
| CPT1A | -1,5493772 | 2,704E-08 |
| LCP1 | -1,4606835 | 3,044E-05 |
| THY1 | -1,4440417 | 1,1259E-06 |
| C3 | -1,3513745 | 5,6331E-06 |
| ABCB5 | -1,3061963 | 0,00024186 |
| NR1H4 | -1,2872162 | 1,2428E-05 |
| NPAS2 | -1,2807504 | 1,4478E-09 |
| KHDRBS3 | -1,2806112 | 3,1426E-08 |
| SGIP1 | -1,1928716 | 1,8136E-07 |
| ADAMTS9 | -1,1748411 | 1,4828E-06 |
| CACNA2D3 | -1,1701907 | 1,0215E-07 |
| HS3ST2 | -1,1226335 | 0,00024107 |
| CLDN10 | -1,0989468 | 0,00021468 |
| GREM1 | -1,0892441 | 0,00012516 |
| MLPH | -1,0785273 | 5,6162E-08 |
| CDH13 | -1,0721972 | 0,00017979 |

Table S6. Top 20 up-regulated genes by re-introduction of p.(Glu527Asp) ClC-5 mutant

|  | rClC-5 p.(Glu527Asp) vs rClC-5 WT | |
| --- | --- | --- |
| Gene Symbol | logFC | adj.p.val. |
| ZPLD1 | 2,24788773 | 5,697E-10 |
| TNNT1 | 1,90773131 | 1,1518E-08 |
| HLA-DRB1 | 1,79629489 | 8,8178E-09 |
| SELENOP | 1,7741324 | 8,6929E-07 |
| CLDN2 | 1,69304491 | 0,00011843 |
| HLA-DMA | 1,55848284 | 7,3078E-08 |
| GPX3 | 1,52380486 | 3,3773E-06 |
| MAPRE3 | 1,51464658 | 2,8941E-06 |
| NDST3 | 1,49303626 | 5,3534E-07 |
| LINC01508 | 1,45530881 | 6,5544E-05 |
| HLA-DPA1 | 1,44850854 | 1,029E-06 |
| SOSTDC1 | 1,42354969 | 0,00014937 |
| CORO2A | 1,38018793 | 5,8778E-06 |
| PAQR5 | 1,3748296 | 0,00038225 |
| LUCAT1 | 1,31447518 | 3,0936E-06 |
| SLC17A1 | 1,3092692 | 0,00193727 |
| SNAI1 | 1,29662188 | 0,00063583 |
| COBL | 1,29648308 | 3,9939E-06 |
| EFCAB13 | 1,2842711 | 0,00010262 |
| SPNS2 | 1,25758919 | 5,6162E-08 |

Table S7. Top 20 down-regulated genes by re-introduction of p.(Ile524Lys) ClC-5 mutant

|  | rClC-5 p.(Ile524Lys) vs rClC-5 WT | |
| --- | --- | --- |
| Gene Symbol | logFC | adj.p.val. |
| CDH1 | -1,10357 | 0,022233 |
| KRT7 | -0,74643 | 0,044569 |
| CATSPER1 | -0,66144 | 0,022233 |
| CLYBL-AS2 | -0,60521 | 0,044569 |
| SLC38A8 | -0,58758 | 0,044569 |
| CNGA2 | -0,51673 | 0,022233 |

Table S8. Top 20 up-regulated genes by re-introduction of p.(Ile524Lys) ClC-5 mutant

|  | rClC-5 p.(Ile524Lys) vs rClC-5 WT | |
| --- | --- | --- |
| Gene Symbol | logFC | adj.p.val. |
| PREX1 | 1,05644 | 0,022233 |
| CP | 1,031158 | 0,022233 |
| TMEM71 | 0,902159 | 0,022233 |
| STEAP1 | 0,752778 | 0,022233 |
| EXOSC8 | 0,70184 | 0,024853 |
| TCIM | 0,662846 | 0,035405 |
| IQCD | 0,660759 | 0,024853 |
| DYNC2H1 | 0,660272 | 0,02306 |
| LIN54 | 0,655919 | 0,023526 |
| LRTOMT | 0,629516 | 0,022233 |
| ZRANB3 | 0,628185 | 0,024853 |
| PLAG1 | 0,624192 | 0,022233 |
| TNIK | 0,614592 | 0,023526 |
| TEX2 | 0,58021 | 0,024853 |
| MIS12 | 0,572103 | 0,022233 |
| STEAP1B | 0,567719 | 0,044569 |
| NUFIP1 | 0,558783 | 0,022233 |
| PXYLP1 | 0,555539 | 0,044569 |
| CHCHD7 | 0,554923 | 0,022233 |
| RAB38 | 0,55239 | 0,044569 |
